# Insulin signaling and reduced glucocorticoid receptor activity attenuate postprandial gene expression in liver

**DOI:** 10.1371/journal.pbio.2006249

**Published:** 2018-12-10

**Authors:** Adrija Kalvisa, Majken S. Siersbæk, Stine M. Præstholm, Line J. L. Christensen, Ronni Nielsen, Oliver Stohr, Sabine Vettorazzi, Jan Tuckermann, Morris White, Susanne Mandrup, Lars Grøntved

**Affiliations:** 1 Department of Biochemistry and Molecular Biology, VILLUM Center for Bioanalytical Sciences, University of Southern Denmark, Odense, Denmark; 2 Division of Endocrinology, Boston Children's Hospital, Department of Medicine, Harvard Medical School, Boston, Massachusetts, United States of America; 3 Institute of Comparative Molecular Endocrinology, University of Ulm, Ulm, Germany; Duke University, United States of America

## Abstract

Hepatic circadian gene transcription is tightly coupled to feeding behavior, which has a profound impact on metabolic disorders associated with diet-induced obesity. Here, we describe a genomics approach to uncover mechanisms controlling hepatic postprandial gene expression. Combined transcriptomic and cistromic analysis identified hundreds of circadian-regulated genes and enhancers controlled by feeding. Postprandial suppression of enhancer activity was associated with reduced glucocorticoid receptor (GR) and Forkhead box O1 (FOXO1) occupancy of chromatin correlating with reduced serum corticosterone levels and increased serum insulin levels. Despite substantial co-occupancy of feeding-regulated enhancers by GR and FOXO1, selective disruption of corticosteroid and/or insulin signaling resulted in dysregulation of specific postprandial regulated gene programs. In combination, these signaling pathways operate a major part of the genes suppressed by feeding. Importantly, the feeding response was disrupted in diet-induced obese animals, which was associated with dysregulation of several corticosteroid- and insulin-regulated genes, providing mechanistic insights to dysregulated circadian gene transcription associated with obesity.

## Author summary

The liver is an essential organ regulating metabolic homeostasis in response to fluctuations of metabolites induced by daily rhythms of food intake. Homeostasis is maintained by precise dynamic regulation of signaling pathways controlling a wealth of enzymatic reactions involving lipid, bile acid, amino acid and glucose synthesis, storage, and redistribution in hepatocytes. Precise temporal expression of hepatic enzymes is crucial for metabolic homeostasis; a major part of circadian hepatic protein expression is regulated by precisely timed gene transcription. Here, we use a genomics approach to identify genes and regulatory regions of the genome involved in feeding-regulated gene expression. We find that transcription factors acting downstream of glucocorticoid and insulin signaling are enriched at regulatory regions repressed by feeding. Importantly, insulin and glucocorticoid signaling operate to cooperatively control the majority of feeding-mediated gene repression, and these signaling pathways are dysregulated in diet-induced obesity impacting dynamic hepatic gene expression.

## Introduction

Precise temporal expression of hepatic enzymes is crucial for metabolic homeostasis, and a major part of hepatic circadian protein synthesis is regulated by precisely timed gene transcription and mRNA translation [1]. This is controlled by complex interactions between rhythmic endocrine signaling, fluctuations of the body temperature, oscillating metabolites, and intrinsic circadian networks [2]. It is well established that genetic disruption of the intrinsic cellular clock, controlled by transcription factors such as brain and muscle Arnt-like protein (BMAL), circadian locomotor output cycles kaput (CLOCK), RAR-related orphan receptor (ROR), and reverse c-erbA (REVERB), has a profound impact on circadian gene transcription in the liver [3–6]. However, rhythmic gene transcription can be restored by controlled feeding regimens [7,8], emphasizing the significance of a food-entrainable oscillator for circadian hepatic gene transcription [2]. Importantly, obesity-associated conditions such as hepatosteatosis, insulin resistance, and diabetes are linked to disruption of circadian transcriptional networks in the liver [9]. For example, rodents with ad libitum access to a high-fat diet (HFD) tend to eat in the physical active phase (nighttime) as well as in the resting phase (daytime), in contrast to ad libitum chow-fed mice, eating primarily during the night/active phase [10–12]. Ad libitum HFD feeding leads to a striking disruption of rhythmic gene expression in the liver [13,14] that can be prevented by simple night-restricted feeding (NRF) of HFD [15]. This is associated with diminished HFD-induced obesity, insulin resistance, and diabetes [15], emphasizing the importance of continuous circadian feeding–fasting cycles to prevent obesity-associated disorders. Thus, it is crucial to understand the mechanisms controlling diurnal gene expression and to determine the direct impact of feeding.

Housing rodents in a 12-hour light/dark cycle and restricting feeding to the active phase (nighttime) is associated with rhythmic hepatic chromatin remodeling [16], transcription [1,17], translation [1], post-translational modifications [18–20], protein translocation [21], and hepatocyte morphology [22]. Most of these studies used omics-based technology to characterize rhythmicity and genetic disruption of intrinsic circadian regulators to gain comprehensive mechanistic insights to the rhythmic regulation. However, as oscillating molecular processes such as transcription are controlled by a number of additional factors, including feeding–fasting cycles, we lack considerable genome-wide knowledge of regulatory mechanisms controlling rhythmic transcription by factors operating together with the intrinsic molecular clock. To specifically investigate the mechanism by which daily feeding–fasting cycles affect circadian gene expression, we focused on a pre- and postprandial time point at the junction between the light and dark phase of a circadian rhythm. By tampering with food availability, we could isolate a set of circadian-regulated genes and enhancers that were primarily regulated by feeding. Analysis of chromatin accessibility and histone acetylation suggested that the majority of feeding-repressed genes is regulated by changed activity of signaling pathways regulating the glucocorticoid receptor (GR) and Forkhead box O1 (FOXO1). These factors occupy chromatin in the preprandial state, and occupancy is reduced postprandially, leading to reduced enhancer activity. Preprandial injection with dexamethasone (dex) and/or insulin receptor antagonist (S961) demonstrated that the pancreas and the hypothalamic–pituitary–adrenal (HPA) axis regulate specific and overlapping transcriptional programs in the liver, which collectively control a significant part of hepatic circadian gene transcription.

## Results

### A subset of the circadian transcriptome in the liver is directly regulated by feeding

**Fig 1:**
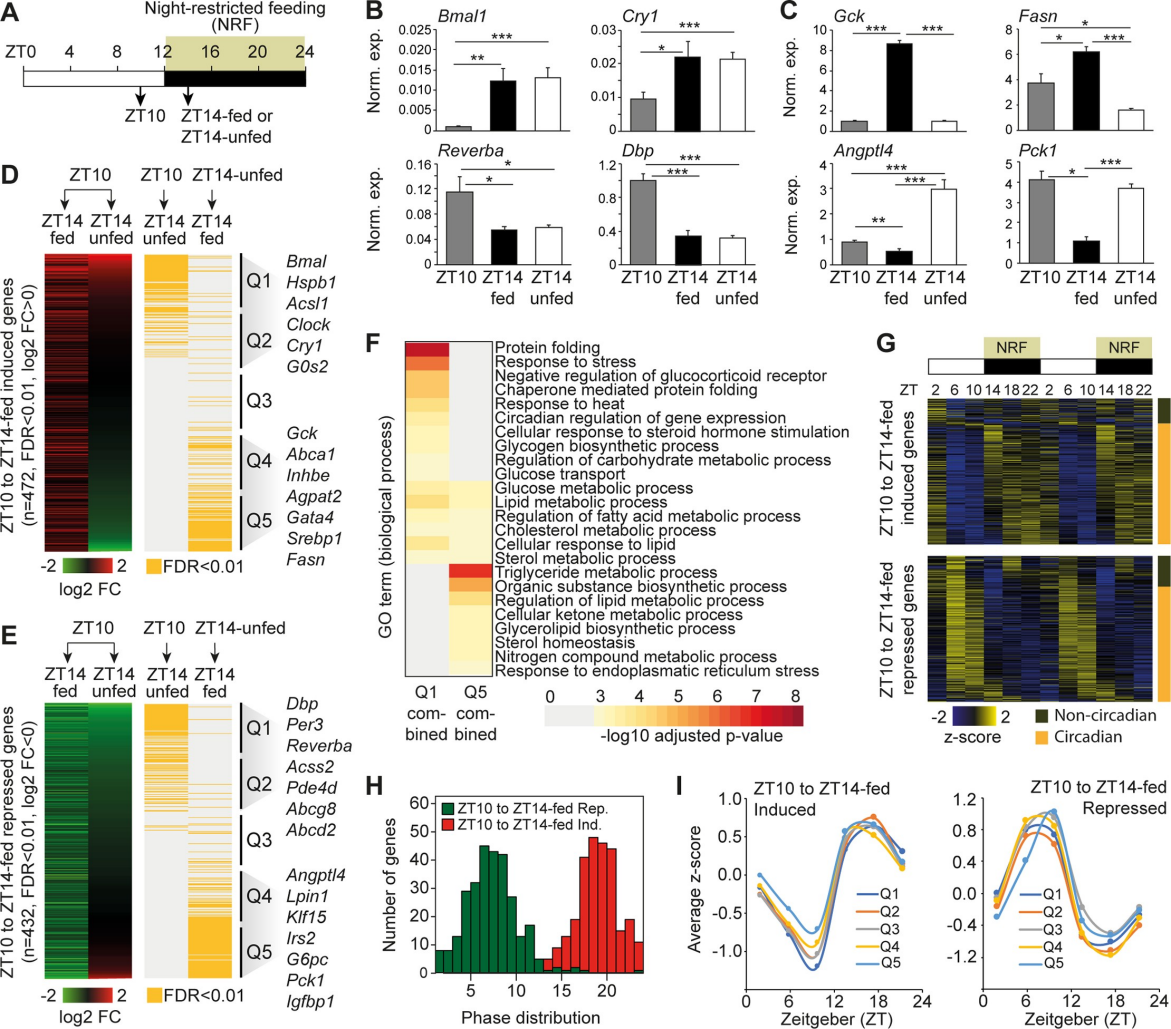
Diurnal hepatic gene expression controlled by feeding. (A) Experimental setup. Mice were housed at constant 12-h light/dark cycles and subjected to NRF. Livers were isolated at ZT10 and ZT14 (ZT14-fed). In parallel, livers from a cohort of mice, kept unfed from ZT12 to ZT14, were isolated at ZT14 (ZT14-unfed). (B) RT-qPCR analysis of genes controlling the core circadian clock and (C) genes regulated by feeding. Data is presented as mean ± SEM (*n* = 5). Statistical significance is evaluated by Student *t* test. (D) Genes induced in the livers from mice at ZT10 compared to ZT14-fed and (E) genes repressed in the livers from mice at ZT10 compared to ZT14-fed. Data is presented as mean log2 FC, FDR < 0.01, *n* = 2. Genes regulated by feeding (ZT14-unfed relative to ZT14-fed) and genes regulated by the transition from ZT10 to ZT14-unfed with FDR < 0.01 are indicated by the orange color scale. Based on the log2 FC (ZT10 relative to ZT14-unfed), genes are grouped into five bins of equal size (Q1–Q5), in which Q1 represents genes primarily unaffected by feeding and Q5 genes primarily affected by feeding. (F) Enriched GO terms for genes in group Q1 and Q5. (G) Circadian expression of genes differentially expressed from ZT10 to ZT14-fed. RPKM values and calculation of phases are from Atger and colleagues [1]. RPKMs are visualized as z-scores. Genes with a phase >1 are indicated with orange. (H) Phase distribution of differentially regulated genes (ZT10 to ZT14-fed). (I) Average z-scores at different time points during a circadian rhythm for genes grouped in Q1–Q5. *p < 0.05, **p < 0.01, ***p < 0.005. Numerical values for panels B–I are available in S1 Data. FC, fold change; FDR, false discovery rate; GO, gene ontology; NRF, night-restricted feeding; RPKM, reads per kilo base per million mapped reads; RT-qPCR, reverse transcription quantitative PCR; ZT, Zeitgeber time.

To specifically evaluate the transcriptional effects of feeding in the liver during a night-restricted experimental setup, we trained mice to NRF. The subsequent experiments were designed to specifically focus on the transition between the resting (lights on, zeitgeber time [ZT]0–ZT12) and the physical active phase (lights off, ZT12–ZT24) with and without access to food (Fig 1A). Livers from night-restricted–fed mice were isolated at ZT10 and ZT14 to monitor the transcriptional effects of feeding and the transition from light to dark. In parallel, isolation of livers from mice (at ZT14) that did not receive food at ZT12 allowed us to analyze the effect of feeding. We initially evaluated mRNA levels of a few genes known to be either controlled by the intrinsic circadian clock or by food intake. All of these genes were regulated by the transition from ZT10 to ZT14-fed (Fig 1B and Fig 1C). When food was omitted, the mRNA levels of the core circadian clock genes *Bmal1*, *Cry1*, *Reverba*, and *Dbp* were not significantly different from levels observed in fed mice. In contrast, the level of mRNAs coding for key metabolic enzymes (glucokinase [GCK], fatty acid synthase [FASN], and phosphoenolpyruvate carboxykinase 1 [PCK1]) and a protein involved in lipoprotein and triglyceride metabolism angiopoietin-like 4 (ANGPTL4) was dependent on food intake (Fig 1C).

To extend this analysis, we performed RNA-seq experiments on livers isolated at ZT10, ZT14-unfed, and ZT14-fed and focused on genes that were induced (*n* = 472) and repressed (*n* = 432) at a false discovery rate (FDR) < 0.01 by the transition from ZT10 to ZT14-fed (Fig 1D and Fig 1E, respectively). The genes in both groups were ranked by their fold change between ZT10 and ZT14-unfed and grouped into five bins of equal size (Q1–Q5). Q1 and Q2 represent genes that were primarily differentially regulated by the transition from ZT10 to ZT14, regardless of food intake, whereas Q4 and Q5 contain genes primarily regulated by food intake. In agreement with the reverse transcription quantitative PCR (RT-qPCR) data (Fig 1B), Q1 and Q2 contain a number of known core circadian clock genes such as *Bmal*, *Per3*, *Cry1*, *Dbp*, *Clock*, and *Reverba*. Moreover, Q1 and Q2 contain genes involved in heat shock response (e.g., *Hspb1*), fatty acid metabolism (e.g., *Acss2*, *Acsl1*, and *Abcd2*), and cholesterol uptake (e.g., *Abcg8*). Q4 and Q5 contain genes such as *Gck*, *Srebp1*, *Fasn*, and *Agpat2* (up-regulated by feeding) involved in glucose uptake and fatty acid synthesis and storage and genes such as *Pck1* and *G6pc* involved in gluconeogenesis (down-regulated by feeding). Gene ontology (GO) analysis showed that genes regulated independent of feeding are involved in protein folding, response to heat, and circadian regulation of gene expression (Fig 1F). In addition, GO analysis also identified enriched biological pathways involved in processes such as glucose, glycogen, fatty acid, and cholesterol metabolism. Some of these metabolic pathways were also enriched in the group of genes regulated by feeding. However, some pathways such as triglyceride and ketone body metabolism were specifically enriched in the genes regulated by feeding (Fig 1F).

To determine whether the different groups of genes were expressed dynamically throughout a circadian rhythm, we compared our data with previously published circadian RNA-seq data from mice subjected to NRF [1]. More than 80% of the genes, identified by us to be differentially expressed from ZT10 to ZT14-fed, were also expressed in a circadian manner (Fig 1G), and most induced and repressed genes had circadian expression zenith at ZT16–21 and ZT5–10, respectively (Fig 1H). Moreover, average expression analysis of the mRNAs in the different gene groups, defined in Fig 1D and 1E (Q1–Q5), suggested that the circadian expression pattern is very similar between genes regulated by feeding (Q4 and Q5) and genes regulated independent of feeding (Q1 and Q2) (Fig 1I). Thus, a large number of circadian-regulated genes are controlled directly by feeding, indicating that the diurnal feeding response is an important driver of circadian gene expression in the liver.

### Feeding-regulated chromatin accessibility and H3K27Ac correlate with feeding-regulated transcription

**Fig 2:**
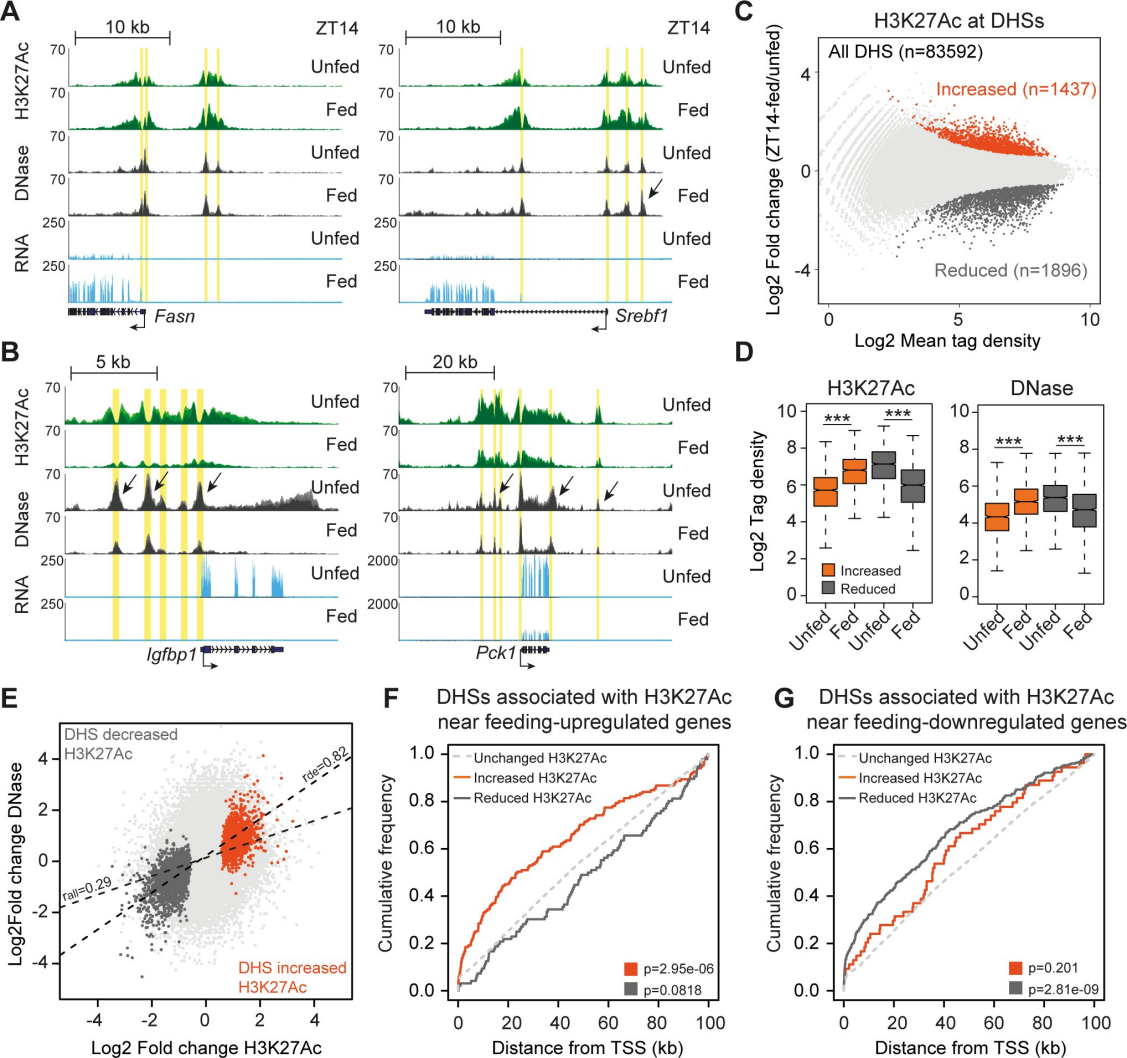
Postprandial DNase accessibility and H3K27 acetylation. RNA-seq, DNase-seq, and H3K27Ac ChIP-seq from livers of mice euthanized at ZT14-unfed and ZT14-fed. (A and B) *Fasn*, *Srebf1*, *Igfbp1*, and *Pck1* mRNA expression and nearby DNase accessibility and H3K27Ac. Yellow regions mark DNase-accessible regions associated with differentially regulated H3K27Ac. Arrows mark differentially regulated DNase accessibility. (C) Differential H3K27Ac at DHSs. Increased H3K27Ac is marked by red, and decreased H3K27Ac is marked by dark gray, FDR < 0.1 (*n* = 3). (D) Tag density of H3K27Ac ChIP-seq and DNase-seq at DHSs associated with feeding-regulated H3K27Ac. Statistical significance is calculated using a Mann–Whitney–Wilcoxon test. ***p > 0.001. (E) Correlation between differentially regulated DNase accessibility and H3K27Ac. Regression analysis is calculated for the differentially regulated DHSs (r_de_) and all DHSs (r_all_) using Pearson correlation. (F) Cumulative distribution of DHSs associated with feeding-regulated H3K27Ac near TSSs of feeding–up-regulated genes. (G) Cumulative distribution of DHSs associated with feeding-regulated H3K27Ac near TSS of feeding–down-regulated genes. A Kolmogorov–Smirnov test was applied to test for differential distribution of DHSs associated with feeding-regulated H3K27Ac (gray and orange line) compared to DHSs associated with no change of H3K27Ac (broken line). Numerical values for panels C–G are available in S2 Data. Bed graphs in panels A and B are available at GEO: GSE119713. ChIP, chromatin immunoprecipitation; DHS, DNase hypersensitive site; FDR, false discovery rate; H3K27Ac, Histone 3 lysine 27 acetylation; TSS, transcription start site.

DNase-accessible regions of chromatin harbor putative binding sites for transcription factors regulating gene expression [23]. To identify mechanisms regulating gene expression controlled by feeding, we profiled DNase-accessible regions genome-wide in livers from fed (*n* = 2) and unfed (*n* = 2) mice at ZT14. To account for possible differences in the digestion efficiency of DNase, we sequenced libraries from nuclei digested with two different concentrations of DNase I (60 U and 80 U) and subsequently combined all the sequencing data to identify DNase hypersensitive sites (DHSs) irrespective of treatment. (Correlation between biological replicates is shown in S1 Fig) Using this approach, we identified a total of 83,592 DHSs, in agreement with other DNase-seq experiments in mouse liver tissue [24,25]. To specifically probe activity of these putative regulatory regions, we performed histone 3 lysine 27 acetylation (H3K27Ac) chromatin immunoprecipitation-sequencing (ChIP-seq) on livers isolated from three unfed (ZT14-unfed) and three fed (ZT14-fed) animals. (Correlation between biological replicates is shown in S2 Fig) For example, most of the DHSs identified near the genes *Fasn*, *Srebf1*, *Igfbp1*, and *Pck1* did change histone acetylation level in response to feeding (Fig 2A and 2B). Genome-wide quantification of H3K27Ac identified a little less than 1,500 DHSs associated with increased H3K27Ac and around 1,900 DHSs associated with decreased H3K27Ac at FDR < 0.1, *n* = 3 (Fig 2C). DNase accessibility at these differentially acetylated DHSs was also changed in response to feeding (Fig 2D), and we observed a genome-wide correlation between changed H3K27Ac and DNase accessibility of the DHSs associated with feeding-regulated H3K27Ac (r_de_ = 0.82, Fig 2E). The correlation between DNase accessibility and H3K27Ac was weaker for all identified DHSs (r_all_ = 0.29). In addition, we found enrichment of DHSs associated with feeding–up-regulated H3K27Ac near feeding-induced genes compared to DHSs associated with unchanged H3K27Ac (Fig 2F, *p* = 2.95 × 10^−06^, Kolmogorov–Smirnov test). Likewise, DHSs associated with feeding–down-regulated H3K27Ac was enriched near feeding-repressed genes compared to DHSs associated with unchanged H3K27Ac (Fig 2G, *p* = 2.81 × 10^−09^, Kolmogorov–Smirnov test). Collectively, this indicates that the identified DHSs are involved in regulating nearby gene expression, and to uncover transcription factors involved in the feeding response, we mined enriched DNA sequences at DHSs associated with feeding-regulated H3K27Ac.

### Enriched motif analysis of differentially H3K27Ac DHSs reveals specific transcription factor networks regulating diurnal enhancer activity

**Fig 3:**
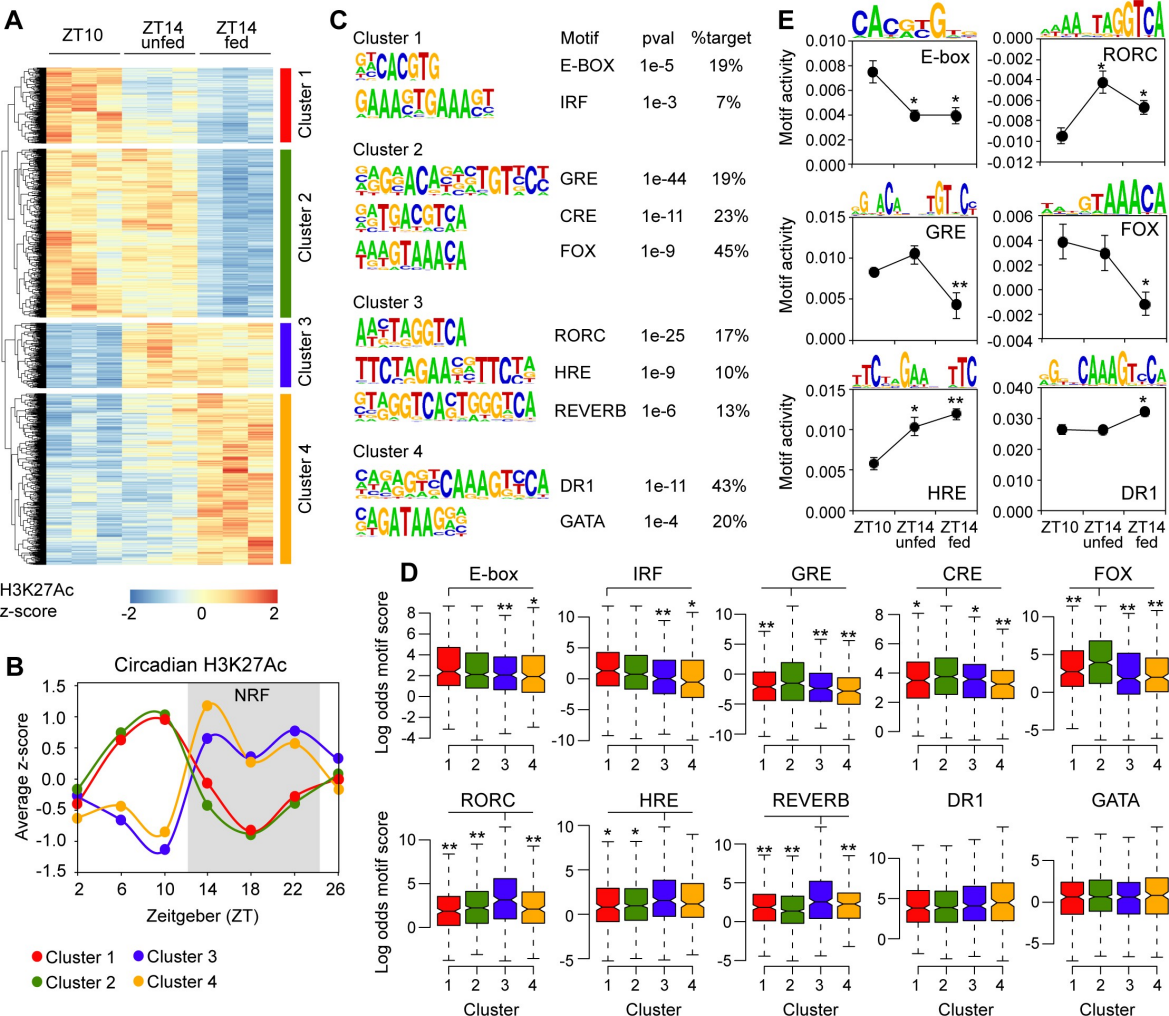
Enriched DNA motif analysis within DHSs associated with differentially regulated H3K27Ac. (A) DHSs differentially H3K27 acetylated at ZT14-fed compared to ZT10 was identified at FDR < 0.1 (*n* = 3). At these DHSs, H3K27Ac was quantified in livers from three biological replicates at ZT10, ZT14-unfed, and ZT14-fed. H3K27Ac tag count is visualized as z-scores. Hierarchical clustering reveals four distinct clusters. (B) Circadian H3K27Ac of DHSs associated with clusters 1–4. Circadian H3K27Ac ChIP-seq data is from livers of NRF animals [16], and the tag count is normalized to a z-score. The average z-score for each cluster is plotted as a function of time. (C) DNA motif analysis of DHSs associated with clusters 1–4. (D) Quantification of the log odds motif score for each motif in DHSs associated with clusters 1–4. Statistical significance is calculated using Kruskal–Wallis test followed by a posthoc pairwise Mann–Whitney–Wilcoxon Test. The Kruskal–Wallis *p*-value is less than 0.05 between clusters for all motifs except DR-1 and GATA. (E) Activity of selected motifs calculated using IMAGE [26]. Data are presented as mean ± SEM (*n* = 3). Statistical significance is calculated using Student *t* test. *p < 0.05 and **p < 0.005. Numerical values for panels A, B, D, and E are available in S3 Data. DHS, DNase hypersensitive site; DR-1, direct repeat; FDR, false discovery rate; H3K27Ac, histone 3 lysine 27 acetylation; IMAGE, integrated analysis of motif activity and gene expression; NRF, night-restricted fed.

To analyze DHSs primarily associated with feeding-regulated H3K27Ac and DHSs mostly associated with H3K27Ac regulated independently of feeding, we first identified DHSs associated with differential H3K27Ac in livers from mice euthanized at ZT10 and ZT14-fed (FDR < 0.1, *n* = 3). H3K27Ac ChIP-seq tags were subsequently quantified at these DHSs from livers isolated at ZT10 (*n* = 3), ZT14-unfed (*n* = 3), and ZT14-fed (*n* = 3) and by hierarchical clustering grouped into two major clusters according to the circadian rhythm and four subclusters according to the feeding status (Fig 3A). Clusters 1 and 3 represent DHSs H3K27 acetylated by processes other than feeding, whereas clusters 2 and 4 were feeding-regulated (Fig 3A). To analyze circadian H3K27Ac at the DHSs differentially H3K27 acetylated between ZT10 and ZT14-fed, we used previously published circadian H3K27Ac ChIP-seq data from livers of mice subjected to NRF [16]. Quantified H3K27Ac at DHSs were normalized and averaged for the four different clusters (Fig 3B). The average H3K27Ac circadian profile of the different clusters were similar (Fig 3B), demonstrating that the feeding-regulated H3K27Ac followed a similar circadian profile as H3K27Ac uncoupled from the feeding response, in agreement with the transcriptomic data (Fig 1I).

To identify putative transcription factors associated with differential H3K27Ac, we searched for enriched DNA motifs from known transcription factor–binding sequences curated by hypergeometric optimization of motif enrichment (HOMER) [27]. The most enriched motifs in clusters 1 and 3 were DNA sequences known to interact with BMAL and CLOCK (binding to E-BOX motifs), RAR-related orphan receptor C (RORC), heat shock factors (heat shock response element [HRE]), and REVERB (Fig 3C). Quantification of the motif score between the different clusters confirmed specific enrichment of the RORC and REVERB motifs in cluster 3 (Fig 3D). The HRE was enriched in cluster 3 compared to clusters 1 and 2; however, no significant difference could be observed between clusters 3 and 4. Also, the E-BOX and interferon regulatory factor (IRF) motifs were enriched in cluster 1 compared to clusters 3 and 4; however, no significant enrichment was observed between cluster 1 and cluster 2 (Fig 3D). To support these findings, we evaluated the contribution of a particular DNA motif to H3K27Ac (referred to as motif activity) of DHSs using IMAGE [26]. This analysis suggested that the E-BOX, HRE, and RORC motifs contributed to differential H3K27Ac in livers from mice at ZT10 compared ZT14, irrespective of feeding (Fig 3E). Thus, DNA motifs shown to interact with transcription factors of the core circadian clock network were specifically enriched in the DHSs associated with differential H3K27Ac uncoupled from feeding.

The DHSs associated primarily with feeding-regulated H3K27Ac (clusters 2 and 4) were enriched for motifs known to interact with the GR, cAMP Responsive Element Binding Protein (CREB), Forkhead box transcription factors (FOX), GATA, and direct repeats (DR-1) known to interact with transcription factors such as Hepatocyte Nuclear Factor 4 (HNF4), Retinoid X receptor (RXR), and peroxisome proliferator-activated receptor (PPAR) [28]. Quantification of the motif score showed that the GR response elements (GRE), CREB response elements (CRE), and FOX motifs were specifically enriched in cluster 2 (Fig 3D), and IMAGE analyses indicated that the GRE and FOX motifs contributed significantly to reduced H3K27Ac in response to feeding (Fig 3E). Moreover, de novo motif analysis of DHSs associated with feeding-regulated H3K27Ac shown in Fig 2C confirmed enrichment of GRE, CRE, and FOX motifs at DHSs associated with feeding-repressed H3K27Ac (S3 Fig).

Collectively, the DNA motif analysis indicated that circadian H3K27Ac is regulated by a combination of core clock transcription factors (e.g., BMAL, CLOCK, and REVERB), heat shock responsive transcription factors, and transcription factors regulated by endocrine signaling (e.g., GR, CREB, and FOXO1).

### DHSs surrounded by feeding-reduced H3K27Ac are occupied by GR, FOXO1, and CREB

To correlate motif enrichment analysis to transcription factor occupancy, we chose to map transcription factors potentially interacting with DHSs associated with feeding-repressed H3K27Ac (Fig 3 and S3 Fig). The identified FOX motifs interact with a range of FOX transcription factors, including FOXO1, known to be negatively regulated by insulin signaling by mechanisms including nuclear exclusion and interaction with transcriptional coregulators [29]. Thus, we mapped FOXO1 together with GR, known to interact with the enriched GRE (Fig 3 and S3 Fig). The CRE motif interacts with CREB, which is post-translationally regulated by glucagon signaling. CREB occupancy of chromatin has been reported previously in 24-hour fasted livers [30,31], and we used these data for analysis of CREB occupancy of chromatin.

**Fig 4:**
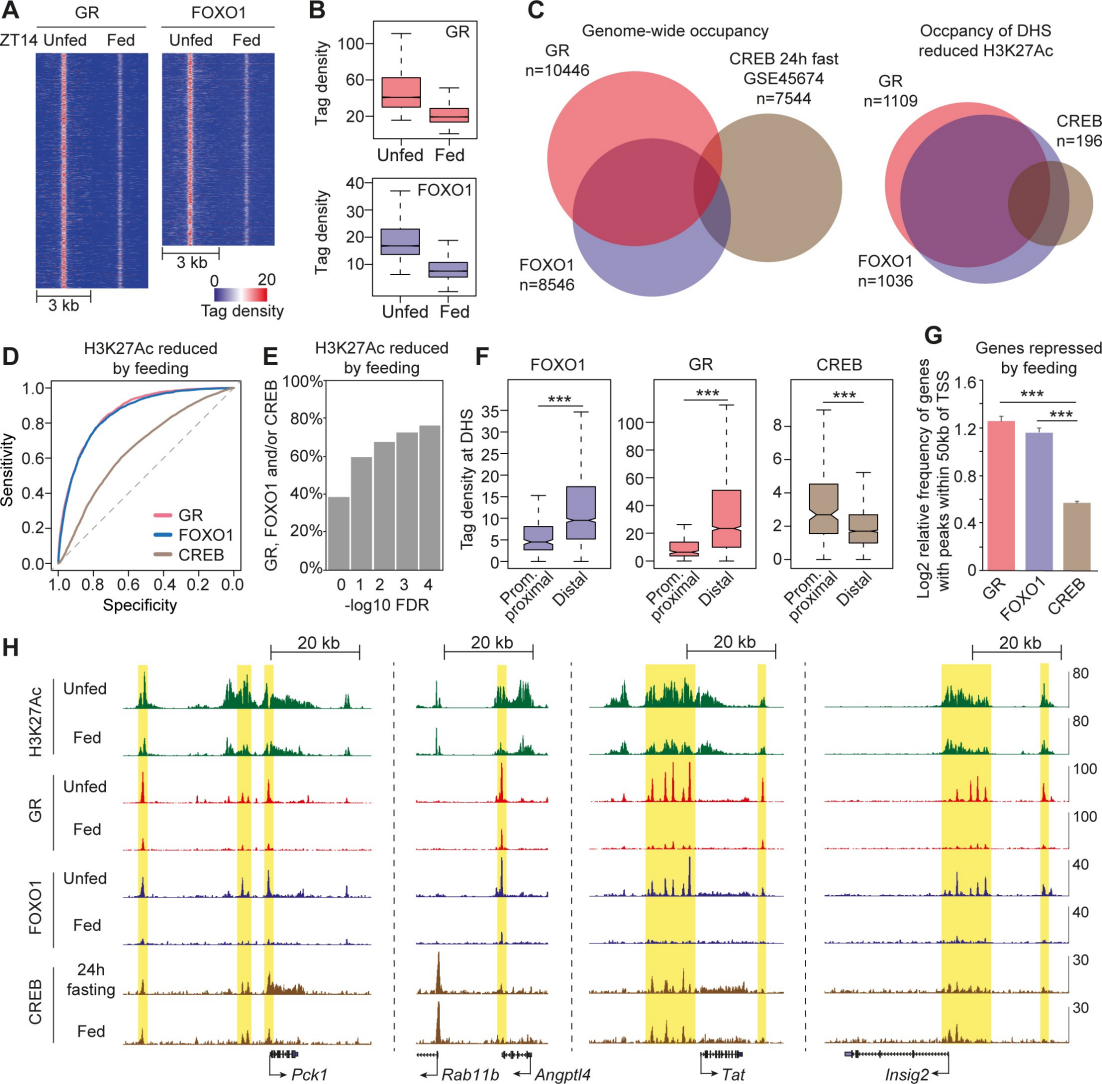
Pre- and postprandial occupancy of GR and FOXO1. GR and FOXO1 ChIP-seq was performed on livers isolated from mice euthanized at ZT14-unfed and ZT14-fed. Replicate concordant GR and FOXO1 peaks were identified in unfed mice. (A and B) Tag density of hepatic GR (*n* = 10,446) and FOXO1 (*n* = 8,546) ChIP-seq in unfed and fed conditions. (C) Co-occupancy of GR, FOXO1, and CREB genome wide (left) and at DHSs surrounded by feeding-reduced H3K27Ac (right). CREB ChIP-seq data is from Everett and colleagues [31]. (D) ROC analysis of GR, FOXO1, and CREB occupancy within DHS surrounded by H3K27Ac reduced by feeding at FDR < 0.05. (E) Percentage of DHSs surrounded by feeding-reduced H3K27Ac occupied by GR, FOXO1, and CREB. DHSs associated with feeding-regulated H3K27Ac was scored at different FDRs (*n* = 3). (F) Tag density of FOXO1, GR, and CREB ChIP-seq at DHSs surrounded by feeding-reduced H3K27Ac located promoter proximal (1 kb upstream the TSS) or distal to the TSS (>1 kb from TSS). Statistical significance is calculated using a Mann–Whitney–Wilcoxon test. (G) Frequency of feeding-repressed genes with at least one GR, FOXO1, or CREB peak within 50 kb of the TSS. Frequency is relative to GR, FOXO1, and CREB binding within 50 kb of TSS of sets of 300 randomly selected genes. Data are presented as mean ± SEM (*n* = 6). Statistical significance is calculated using Student *t* test. (H) Examples of GR, FOXO1, and CREB enrichment within enhancers repressed by feeding. ***p < 0.001. Numerical values for panels A–G are available in S4 Data. Bed graphs in panel G are available at GEO: GSE119713. ChIP, chromatin immunoprecipitation; CREB, cAMP responsive element binding protein; DHS, DNase hypersensitive site; FDR, false discovery rate; FOXO1, Forkhead box O1; GR, glucocorticoid receptor; H3K27Ac, Histone 3 lysine 27 acetylation; ROC, receiver operating characteristic; TSS, transcription start site.

ChIP-seq identified more than 10,000 GR peaks and more than 8,000 FOXO1 peaks in livers from unfed mice at ZT14. De novo motif analysis of the individual transcription factor ChIP-seq peaks identified their cognate DNA-binding motif in addition to motifs for the linage-determining transcription factors HNF4 and C/EBP (S4A Fig), suggesting that the ChIPs enrich for GR and FOXO1-binding sites. Moreover, the specificity of the FOXO1 antibody used for ChIP was validated by western blotting (S4B Fig). Feeding resulted in significant genome-wide reduction of GR and FOXO1 occupancy (Fig 4A and 4B). Interestingly, in unfed livers, GR co-occupied more than 60% of FOXO1 binding sites genome wide, whereas little overlap was observed between CREB and GR–FOXO1 (Fig 4C, left). This pattern of co-occupancy was observed irrespective of the CREB data set used for the analysis (S4C Fig). Strikingly, FOXO1 and GR co-occupancy was even more pronounced at DHSs associated with reduced H3K27Ac in response to feeding, as defined in Fig 2C (Fig 4C, right). Furthermore, CREB occupancy of these regions is mostly associated with co-occupancy of GR and/or FOXO1. Receiver operating characteristic (ROC) analysis suggested that the level of GR and FOXO1 occupancy in unfed conditions are better predictors of feeding-reduced H3K27Ac compared to CREB (Fig 4D). This suggests that loss of H3K27Ac in response to feeding is primarily caused by reduced GR and FOXO1 occupancy. More than 60% of the DHSs associated with feeding-reduced H3K27Ac were occupied by GR, FOXO1, and/or CREB. If we decreased the FDR for calling differentially regulated H3K27Ac, we observed an increased frequency of GR, FOXO1, or CREB occupancy (Fig 4E), suggesting that DHSs associated with the most robustly feeding-regulated H3K27Ac were more likely occupied by at least one of those three transcription factors. GR and FOXO1 occupancy were most pronounced at distal DHSs repressed by feeding (Fig 4F), whereas CREB preferentially occupied DHSs in the proximal promoter regions (transcription start site [TSS] +/− 2 kb).

Correlation with feeding-regulated genes demonstrated that more than 80% of the feeding-repressed genes were associated with at least one GR and/or FOXO1-binding site within 50 kb of the TSS (Fig 4G), corresponding to more than 2-fold enrichment compared to random genes in the genome. Interestingly, a relative high frequency of feeding-induced genes was also associated with at least one GR and/or FOXO1-binding site within 50 kb of the TSS (S4D Fig); however, the enrichment of GR and/or FOXO1 near feeding-induced genes relative to random genes was less pronounced compared to feeding-repressed genes. Moreover, the number of GR and FOXO1 peaks (S4E Fig) and the tag density (S4F Fig) of the peaks near feeding-induced genes were significantly lower compared to GR and FOXO1 peaks identified near feeding-repressed genes. We also observed higher enrichment of feeding-repressed genes with at least one CREB-binding site within 50 kb of the TSS compared to feeding-induced genes (Fig 4F). However, we could not observe any difference in the tag density of CREB peaks (S4F Fig) and the amount of CREB peaks (S4E Fig) near feeding-repressed genes compared to feeding-induced genes. Fig 4H illustrates examples of GR, FOXO1, and CREB occupancy near genes repressed by feeding (*Pck1*, *Angptl4*, *Tat*, and *Insig2*). Notice that most regions are associated with considerable co-occupancy of GR and FOXO1.

In summary, these data indicate that a large fraction of DHSs associated with feeding-repressed H3K27Ac are occupied by GR and FOXO1 in the preprandial state. Feeding leads to reduced occupancy of GR and FOXO1, possibly resulting in postprandial repression H3K27Ac and attenuated transcription of nearby genes.

### Glucocorticoids control H3K27Ac at a subset of feeding-regulated DHSs

**Fig 5:**
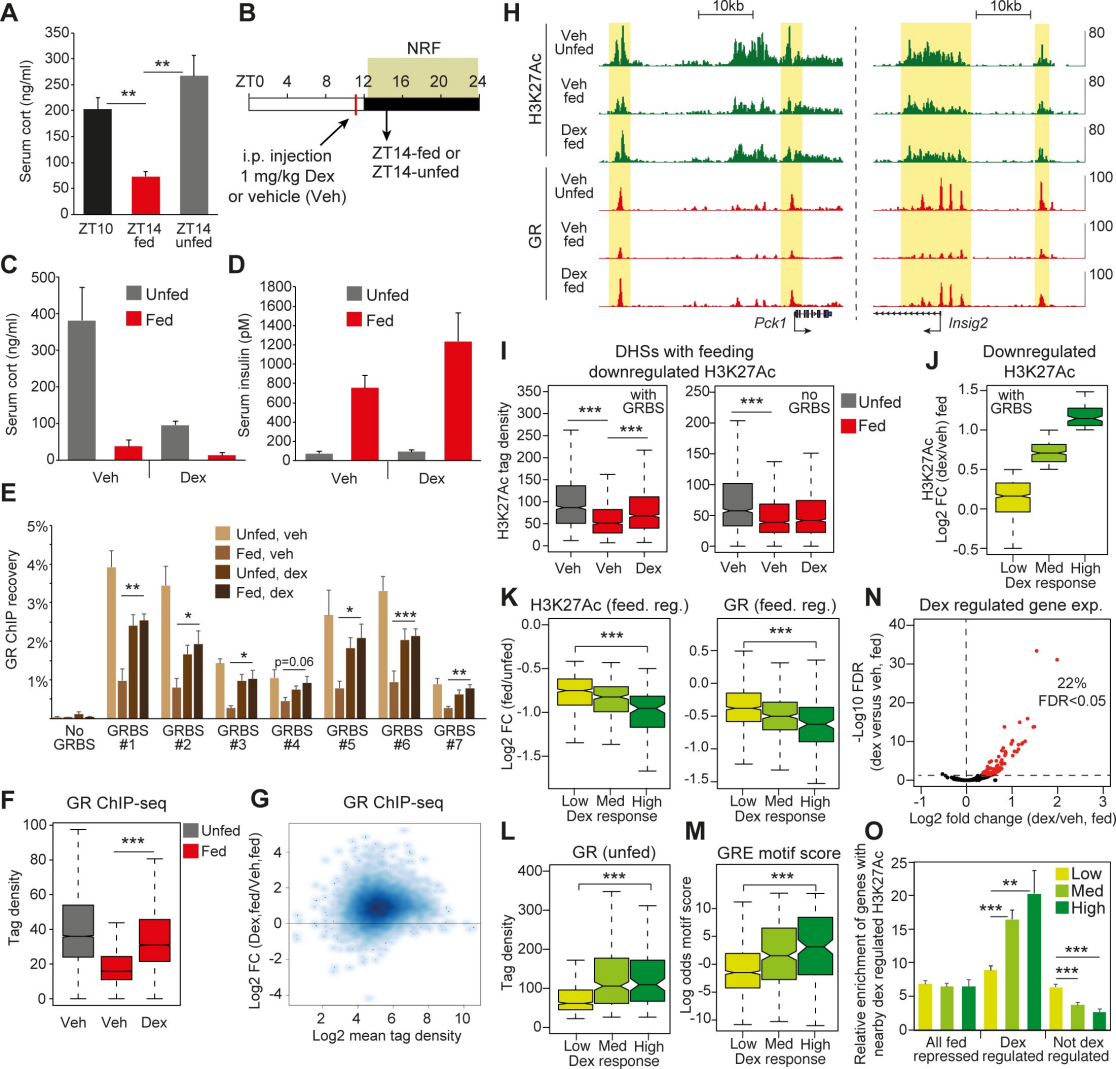
Regulation of postprandial enhancer activity by GR. (A) Corticosterone levels in serum from mice euthanized at ZT10, ZT14-fed, and ZT14-unfed. (B) Preprandial dex (or vehicle) injection protocol for animals trained to NRF. Animals were euthanized at ZT14-fed and ZT14-unfed. (C) Corticosterone and (D) insulin levels in serum of animals injected with dex or vehicle. (E) GR occupancy of seven different GRBS and one control site (no GRBS). (F) Genome-wide occupancy of GR in livers isolated from mice euthanized at ZT14-unfed (vehicle injected) and ZT14-fed (vehicle or dex injected). (G) Effect of dex injection on genome-wide GR occupancy in fed animals at ZT14. (H) Dex-regulated GR occupancy and H3K27Ac near *Pck1* and *Insig2*. Yellow marks H3K27Ac regions occupied by GR. (I) Effect of dex injection on H3K27Ac at feeding-repressed enhancers with and without a GRBS. (J) Effect of dex injection on H3K27Ac at feeding-repressed enhancers with a GRBS. Enhancers are grouped into three bins based on differential H3K27Ac levels in response to dex. (K) Feed. reg. H3K27 acetylation (left) and GR occupancy (right) of feeding-repressed enhancers acetylated to a different degree by dex injection. (L) Level of GR occupancy of enhancers with differentially H3K27Ac after dex injection. (M) GRE motif score within enhancers differentially H3K27Ac by dex. (N) Differential expression of feeding-repressed genes affected by dex injection compared to animals injected with vehicle (quantified by RNA-seq). Red marks genes induced by dex at FDR < 0.05 (*n* = 4 in each treatment group). (O) Relative enrichment of feeding-repressed genes (dex-regulated or not) harboring dex-regulated enhancers within 50 kb of the TSS. Enrichment is relative to six sets of 400 randomly selected genes. Data in panels A, C, D, E, and N are presented as mean ± SEM (*n* = 4–6). Statistical significance is calculated using Student *t* test. Statistical analysis in panels F, I–M is performed by a Mann–Whitney–Wilcoxon test. *p < 0.05, **p < 0.01, ***p < 0.001. Numerical values for panels A, C–G, and I–O are available in S5 Data. Bed graphs in panel H are available at GEO: GSE119713. dex, dexamethasone; feed. reg., feeding-regulated; FDR, false discovery rate; GR, glucocorticoid receptor; GRBS, GR binding site; NRF, night-restricted feeding; TSS, transcription start site; ZT, Zeitgeber time.

Recruitment of GR to chromatin and subsequent chromatin remodeling is strictly dependent on the glucocorticoid level in the surrounding environment [32,33]. In ad libitum–fed rodents, corticosterone levels surge toward the end of the resting phase (zenith at ZT10-ZT12) when the animals are fasting and declines when animals enter the physical active/dark phase [34]. Thus, postprandial reduction of GR occupancy of the genome is possibly mediated by reduced levels of circulating corticosterone as a result of feeding. Indeed, in our experimental setting, feeding resulted in reduced corticosterone levels in serum (Fig 5A). To determine the significance of the reduced levels of glucocorticoids, we injected dex or vehicle immediately before feeding to short circuit the feeding response and collected livers at ZT14 from fed or unfed animals (Fig 5B). GR occupancy and H3K27Ac were subsequently probed by ChIP at ZT14 in unfed and fed conditions. Importantly, control injections (vehicle) did not perturb feeding-repressed corticosterone levels and feeding-induced insulin levels (Fig 5C and 5D), suggesting that the injection protocol did not interfere with the feeding response. Injection of dex augmented postprandial GR occupancy of several GR-binding sites in the genome (Fig 5E) tested by ChIP-qPCR, and genome-wide analysis indicated that the vast majority of GR binding to chromatin was increased in the fed state after dex injection (Fig 5F and 5G). For example, suppression of GR occupancy near *Pck1* and *Insig2* in response to feeding was reversed by dex injection (Fig 5H).

To identify the functional significance of altered glucocorticoid levels, we quantified H3K27Ac at DHSs (with and without GR occupancy) after dex injection. We observed a general trend of increased H3K27Ac at the DHSs occupied by GR, in contrast to DHSs not occupied by GR (Fig 5I). This suggests that the variation of glucocorticoid concentrations leads to dynamic regulation of H3K27Ac at DHSs occupied by GR. Interestingly, however, not all GR occupied DHSs were acetylated at H3K27 in response to dex injection. For example, a GR-binding site upstream of *Pck1* was H3K27 acetylated in response to dex, whereas H3K27 acetylation of the GR binding site in the proximal promoter was largely unaffected (Fig 5H). Similarly, feeding-repressed H3K27 acetylation of GR-binding sites near *Insig2* persisted after dex treatment (Fig 5H), indicating that a subset of GR-occupied enhancers was unresponsive to dynamic glucocorticoid signaling.

To systematically compare the constituents of DHSs associated with differential H3K27Ac in response to glucocorticoid treatment, we divided DHSs occupied by GR into three groups depending on the H3K27Ac responsiveness to dex (Fig 5J). The DHSs associated with most pronounced increase in H3K27Ac in response to dex were also associated with a significant reduction of H3K27Ac and GR occupancy in response by feeding (Fig 5K). This analysis was confirmed by a linear correlation analysis showing a negative correlation between increased H3K27Ac in response to dex and reduction of H3K27Ac and GR occupancy in response to feeding (S5A and S5B Fig). Moreover, dex-induced H3K27Ac correlated positively with the level of GR occupancy (Fig 5L and S5C Fig) and the GR motif strength (Fig 5M and S5D Fig). In agreement, the level of GR occupancy and the GRE motif strength are better predictors of dex-induced H3K27Ac compared to FOXO1 and CREB occupancy and presence of their respective motifs (S5E Fig). Interestingly, GR occupied DHSs associated with low H3K27Ac in response to dex showed prominent FOXO1 and CREB occupancy (S6A and S6B Fig), indicating that these transcription factors may contribute to feeding-regulated H3K27Ac of the weak GR-binding sites.

Feeding-repressed genes are enriched for nearby DHSs associated with reduced H3K27Ac, which is functionally linked to GR occupancy and dynamic glucocorticoid levels (Fig 4D and Fig 5K), suggesting that GR is a putative regulator of feeding-controlled gene transcription. However, since GR binding per se is not indicative of feeding-regulated H3K27Ac, it is likely that many genes, harboring DHSs occupied by GR, are nonresponsive to circulating corticosteroids. To test this, we evaluated expression of genes harboring nearby occupancy of GR. Feeding-mediated repression of *Tat*, *Fkbp5*, and *Pck1* expression was restored or partially restored upon dex treatment (Fig 5H and S6D and S6E Fig), whereas dex did not reestablish expression of *Angptl4*, *Insig2*, and *G6pc* (Fig 5H and S6F and S6G Fig), despite considerable nearby occupancy of GR. Genome-wide analysis by RNA-seq showed that dex rescued expression of 22% of the feeding-repressed genes at FDR < 0.05 (Fig 5N), demonstrating that dynamic glucocorticoid levels controlled a subset of genes regulated by feeding. To link dynamic gene expression with H3K27Ac at DHSs, we quantified the number of feeding-regulated genes harboring nearby DHSs associated with dex-regulated H3K27Ac (high, medium, and low) and compared this to analysis of random selected genes (i.e., relative gene enrichment). Relative enrichment of genes with nearby occupancy of dex-induced H3K27Ac was particularly evident for genes regulated by dex (Fig 5O). Genes not regulated by dex were less enriched for nearby dex-induced H3K27Ac at DHSs.

Collectively, this demonstrates that reduced levels of circulating corticosterone controls genome-wide GR occupancy of chromatin. Subsequent regulation of H3K27Ac depends on the strength of the underlying GRE and level of GR occupancy and ultimately determines the regulation of nearby target genes. Expression of genes associated with glucocorticoid-regulated H3K27Ac at DHSs was more likely controlled by circulating glucocorticoids.

### Postprandial transcriptional attenuation is regulated by cooperative action of corticosteroid and insulin signaling

**Fig 6:**
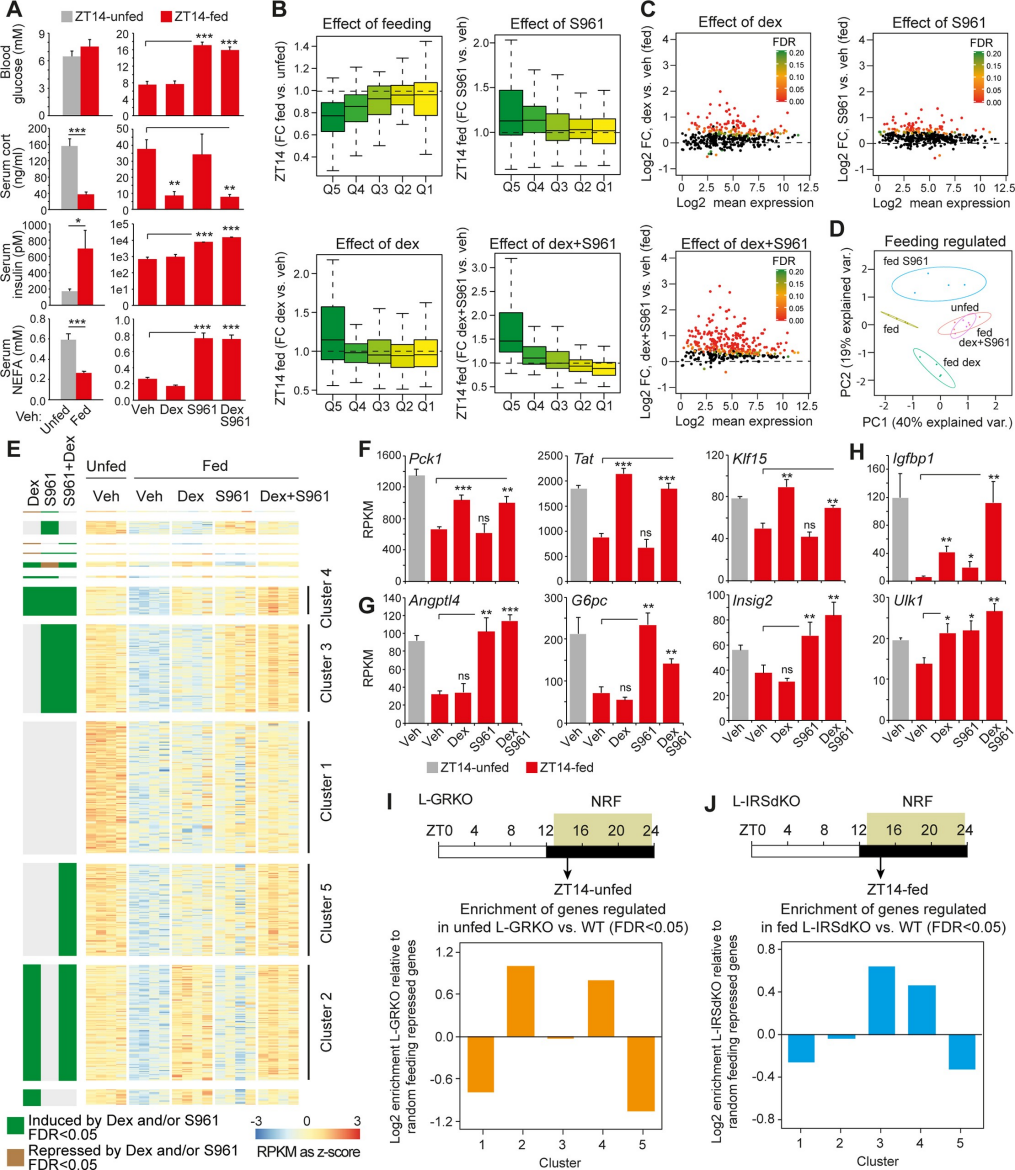
Regulation of postprandial gene expression by glucocorticoid and insulin signaling. (A) Blood glucose and corticosterone, insulin and NEFA levels in serum from mice injected preprandial with dex and/or S961. Data are presented as mean ± SEM (*n* = 4). Statistical significance is calculated using Student *t* test. (B) Relative expression of genes (repressed in ZT14-fed animals compared to ZT10-unfed animals) in livers isolated from animals injected preprandial with vehicle, dex, S961, and dex + S961 (average FC of RPKM, *n* = 4). Relative expression is calculated for fed versus unfed animals (vehicle injected) and fed animals injected with vehicle versus fed animals injected with S961, dex, and dex + S961, respectively. Data are plotted for gene repressed from the ZT10 to ZT14-fed (Q1–Q5) characterized in Fig 1. (C) Differential expression of feeding-repressed genes regulated by dex, S961, and dex + S961 injection. Three hundred sixty-nine feeding-repressed genes were identified from ZT14-unfed versus fed vehicle injected animals at FDR < 0.05 (*n* = 4). Differential expression of these feeding-regulated genes was subsequently visualized from fed animals injected preprandial with vehicle, dex, S961, and dex + S961 injections. FDR is indicated for individual feeding-repressed genes for which black represents FDR > 0.2 (*n* = 4 in each treatment group). (D) Principle component analysis (log2 RPKM) of feeding-regulated genes (identified at FDR < 0.01) in livers from unfed and fed mice injected with vehicle and fed mice injected preprandial with dex, S961, or dex + S961. Circles represent error ellipses at 0.9. (E) Isolation of feeding-repressed genes (identified at FDR < 0.05 and log2 FC < 0) regulated by dex (cluster 2), S961 (cluster 3), dex + S961 (cluster 5), and by both dex and S961 (cluster 4) at FDR < 0.05 (*n* = 4). Cluster 1 represents feeding-repressed genes not regulated by dex and S961. (F, G, and H) Examples of genes in clusters 2, 3, 4, and 5. RPKM is presented as mean ± SEM (*n* = 4). Statistical significance is calculated using Student *t* test, *p < 0.01, **p < 0.005, ***p < 0.001, and ns indicates *p* > 0.05. (I) Enrichment of genes in clusters 1–5 regulated by liver-specific disruption of GR (L-GRKO). Differentially expressed genes in L-GRKO were identified at FDR < 0.05 (*n* = 4), and enrichment of these GR regulated genes in clusters 1–5 was subsequently evaluated relative to randomly selected feeding-regulated genes. (J) Enrichment of genes in clusters 1–5 regulated by liver-specific disruption of IRS1/2 (L-IRSdKO). Differentially expressed genes in L-IRSdKO were identified at FDR < 0.05 (*n* = 4), and enrichment of these insulin receptor-regulated genes in clusters 1–5 was subsequently evaluated relative to 100 randomly selected feeding-regulated genes. Numerical values for panels A–J are available in S6 Data. dex, dexamethasone; FC, fold change; FDR, false discovery rate; GR, glucocorticoid receptor; IRS1, Insulin Receptor Substrate 1; L-IRSdKO, liver specific insulin receptor substrate knockout; L-GRKO, liver specific GR knockout; NEFA, nonesterified fatty acid; RPKM, reads per kilo base per million mapped reads; ZT, Zeitgeber time

It is evident from the above analysis that feeding-repressed gene expression is regulated partly by dynamic GR signaling, and additional signaling pathways regulating transcription factors such as FOXO1 and CREB activity are involved. To address the importance of insulin signaling, we injected insulin receptor antagonist S961 immediately before feeding and isolated livers two hours after feeding at ZT14 (S7A Fig). Treatment with S961 resulted in hyperglycemia and hyperinsulinemia but did not affect corticosterone levels (Fig 6A), indicating that circulating insulin and corticosterone levels are regulated independently of each other. Moreover, acute disruption of insulin signaling resulted in elevated levels of postprandial nonesterified fatty acids (NEFAs), likely as a result of perturbed insulin-mediated repression of adipocyte lipolysis. Interestingly, expression of the dex-unresponsive genes, *Angptl4*, *Insig2*, and *G6pc* (S6C Fig), was restored in the liver of fed animals injected with S961, whereas expression of the dex-responsive genes *Tat*, *Pck1*, and *Fkbp5* was unaffected by S961 (S6C Fig). These results indicate that insulin signaling regulates expression of a subset of feeding-regulated genes, whereas glucocorticoids preferentially control expression of other feeding-regulated genes.

To characterize these transcriptional programs genome wide, we performed RNA-seq on livers from unfed and fed mice injected preprandial with S961 and S961 together with dex (*n* = 4 for all treatment groups). Coinjection resulted in hyperglycemia and hyperinsulinemia and suppressed corticosterone levels as a result of negative feedback inhibition of the HPA axis (Fig 6A). Initially, we evaluated the acute pharmacological effect on RNA expression of the clustered circadian-regulated genes (Q1–Q5, down-regulated from ZT10 to ZT14-fed) defined in Fig 1E. In agreement with the initial separation of circadian-regulated genes into feeding and nonfeeding-regulated, we observed that genes primarily down-regulated by the intrinsic circadian program were not affected by feeding (Q1 and Q2) (Fig 6B). In contrast, the mRNA levels of genes down-regulated by the feeding response (Q4 and Q5) were reduced in response to feeding. Treatment with S961 or dex resulted in increased expression of feeding down-regulated genes (Q4 and/or Q5) in fed condition, whereas intrinsic circadian-regulated genes were unaffected (Q1 and Q2). This was augmented when animals were treated with dex in combination with S961. To illustrate the cooperative effect of dex and S961 on feeding-repressed genes, we identified all feeding-repressed genes in mice injected with vehicle (at FDR < 0.05, 369 genes were suppressed by feeding) and, in parallel, analyzed differential mRNA expression in response to dex, S961, and dex + S961. From this analysis, we extracted the feeding-repressed genes and illustrated the response to dex and/or S961 as M (log ratio) and A (mean average) (MA) plots (Fig 6C). This demonstrated that coinjection of S961 and dex resulted in a more pronounced induction of mRNA expression compared to individual treatments. In agreement, principle component analysis showed that the combined action of dex and S961 resembled the mRNA expression pattern of unfed animals (Fig 6D), indicating that the cooperate action of the two separate signaling pathways controls postprandial suppression of gene transcription.

To identify clusters of genes preferentially regulated by dex and/or S961, we identified all feeding-repressed genes and separated genes regulated by S961, dex, and/or dex + S961 at FDR < 0.05. Based on this analysis, we binned the genes into five major clusters. Cluster 1 did not show any statistically significant response to treatment (Fig 6E). Two clusters of genes responded either to dex (cluster 2) or S961 (cluster 3), demonstrating that the pancreatic and HPA-signaling pathways to some degree operate specific transcriptional programs in the liver. For example, *Pck1*, *Tat*, and *Klf15* were activated primarily by dex, whereas *Angptl4*, *G6pc*, and *Insig2* were regulated preferentially by S961, in agreement with the RT-qPCR data (Fig 6F and 6G, respectively). Moreover, we did observe clusters of genes that were activated by both dex and S961 independently (cluster 4) and when dex and S961 were given in combination (cluster 5), suggesting that insulin and corticosteroid coregulate a subset of genes, either additively or synergistically. This includes genes such as *Igfbp1* and *Ulk1* (Fig 6H).

To verify these findings and to determine liver-specific effects of insulin receptor and GR signaling, we performed similar sets of NRF experiments in mice harboring liver-specific disruption of GR (L-GRKO) (S7B–S7D Fig) and previously characterized mice with liver-specific disruption of Insulin Receptor Substrate 1 (IRS1) and IRS2 (L-IRSdKO) [35]. Quantification of RNA expression by RNA-seq of feeding-repressed genes regulated by dex and/or S961 confirmed that the expression of genes regulated by dex was primarily disrupted in livers from L-GRKO mice (S7E Fig), and the expression of genes regulated by S961 was perturbed in livers from L-IRSdKO mice (S7F Fig). To determine whether any of the clusters described in Fig 6E were predominantly enriched for GR or IRS1/2 regulated genes, we identified differentially regulated genes in the livers of L-GRKO mice and L-IRSdKO mice relative to wild-type (WT) controls at FDR < 0.05, *n* = 4. This demonstrated that the differentially regulated genes in the L-GRKO mice were enriched in cluster 2 and cluster 4 (Fig 6I), corresponding to dex-regulated genes. In contrast, the differentially regulated genes in the L-IRSdKO mice were enriched in cluster 3 and cluster 4, corresponding to S961-regulated genes (Fig 6J). Interestingly, genes synergistically regulated by dex and S961 (cluster 5) were less likely regulated in the L-GRKO model and the L-IRSdKO models (Fig 6I and 6J and S7E and S7F Fig), suggesting that insulin- and corticosterone-mediated regulation of these genes requires both signaling pathways to be intact to maintain regulation. Alternatively, this group of genes may be primarily controlled by signaling through extrahepatic tissues. For example, S961 disrupts feeding-regulated NEFA levels, likely by perturbed insulin-mediated repression of lipolysis in adipose tissue, impacting lipid metabolism and gluconeogenesis in the liver [36], and may also affect hepatic gene expression. In summary, these data indicate that postprandial repression of hepatic gene expression is controlled by cooperative action of insulin and corticosterone signaling.

### Disrupted circadian feeding response in diet-induced obese animals

**Fig 7:**
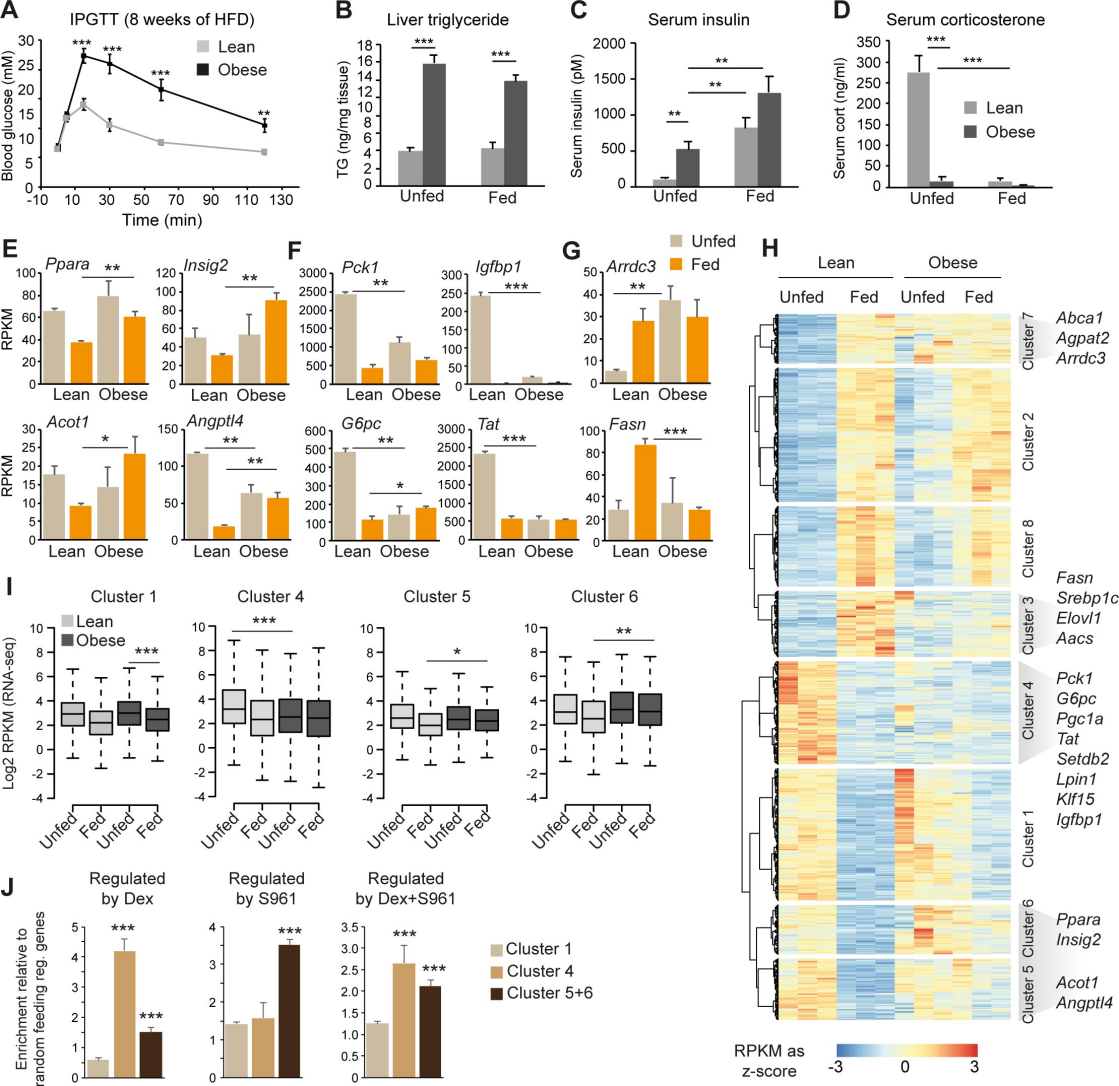
Postprandial gene expression in diet-induced obese mice. Mice were fed chow or HFD for 10 weeks (ad libitum). (A) Glucose tolerance test of chow-fed (*n* = 8) and HFD-fed (*n* = 8) animals after eight weeks on HFD. (B) Liver triglyceride levels, (C) insulin levels, and (D) corticosterone levels in chow-fed (*n* = 6), chow-unfed (*n* = 5), HFD-fed (*n* = 5), and HFD-unfed (*n* = 5) animals. (E–G) Examples of feeding-regulated gene expression in lean and obese animals. (H) Hierarchical clustering analysis of genes regulated by feeding in lean animals at FDR < 0.01 compared to feeding-regulated gene expression in obese animals. Clustering and differential expression analysis was performed using RNA-seq data from three biological replicates in each condition. RPKM values are normalized to a z-score for each individual gene across conditions. (I) Quantification of feeding-repressed genes (clusters 1, 4, 5, and 6) in livers from lean and obese mice. Statistical significance is calculated using a Mann–Whitney–Wilcoxon test on average expression between three biological replicates. (J) Enrichment of genes in clusters 1, 4, and 5 + 6 regulated by dex, S961, or dex + S961 at FDR < 0.01 relative to randomly selected feeding-regulated genes. Each condition is an average of four individual replicates. Data presented in panels A–G and J are plotted as mean ± SEM. Statistical significance is calculated using Student *t* test. *p < 0.05, **p < 0.01, and ***p < 0.001. Numerical values for panels A–H are available in S7 Data. FDR, false discovery rate; HFD, high-fat diet; RPKM, reads per kilo base per million mapped reads

Circadian transcription in liver from diet-induced obese animals is severely disrupted by a number of metabolic pathways interacting with the intrinsic circadian clock machinery [9,37]. It is known that mice on an HFD have disordered feeding patterns and abnormal transcriptional rhythmicity [12–14] and that NRF of HFD reduces obesity and minimizes the risk of developing insulin resistance and diabetes [15]. Thus, the disrupted circadian hepatic gene expression observed in HFD-induced obese animals is likely strongly associated with a disrupted circadian feeding response. To investigate the circadian feeding response in diet-induced obese animals, we fed mice an HFD for 10 weeks. HFD feeding resulted in significant weight gain (S8A Fig) and resulted in reduced glucose tolerance (Fig 7A). Obese and lean control mice were trained to NRF, and livers were subsequently isolated from unfed and fed lean and obese mice at ZT14 (S8B Fig). In agreement with the reduced glucose tolerance, liver triglycerides (Fig 7B) and insulin levels (Fig 7C) were significantly increased in unfed obese animals at ZT14 compared to lean unfed controls. Moreover, preprandial corticosterone levels were severely reduced in unfed obese animals (Fig 7D) compared to lean controls. This effect was independent of the duration of HFD feeding (S8C Fig).

To examine the effect of HFD on feeding-regulated gene expression in liver, we performed RNA-seq on livers isolated from unfed and fed lean and obese animals at ZT14. Although RNA expression analysis demonstrated disrupted feeding-regulated expression of a number of genes (Fig 7E–7G), the cause of impaired feeding response was gene specific. For example, repression of *Ppara*, *Insig2*, *Acot1*, and *Angptl4* expression by feeding in lean animals was decreased in obese animals as a result of elevated mRNA expression in the fed obese animals compared to lean controls (Fig 7E). And expression of *Pck1*, *G6pc*, *Igfbp1*, and *Tat* was significantly decreased in unfed obese mice compared to lean controls, leading to a reduced feeding response (Fig 7F). A similar tendency could be observed for feeding-induced gene expression. For example, feeding-induced expression of *Arrdc3* was blunted as a result of increased expression in the liver of unfed obese animals, whereas feeding-induced *Fasn* expression was impaired as a result of reduced *Fasn* expression in fed obese animals (Fig 7G).

To probe genome-wide effects on gene expression, we identified all differentially regulated genes in response to feeding in chow-fed animals at FDR < 0.01 (*n* = 3) and subsequently performed hierarchical clustering of all biological replicates (*n* = 3 for each condition) from fed and unfed chow and diet-induced obese animals. This approach is independent of a specific FDR cutoff, which allowed us to group genes despite some variability between biological replicates. Hierarchical clustering revealed eight distinct clusters of feeding–up-regulated and feeding–down-regulated genes (Fig 7H), and differential gene expression analysis was performed to confirm disrupted feeding response of specific clusters (S8D Fig). Five of these gene clusters (3, 4, 5, 6, and 7) showed clear impaired feeding response in obese animals (Fig 7H and S8D Fig). Two clusters of feeding-induced genes demonstrated an impaired feeding response with seemingly different mechanisms. Expression of genes in cluster 3, including the transcription factor *Srebp1c* and its target genes *Fasn*, *Aacs*, *Sqle*, and *Ldlr*, was reduced in the liver of fed obese animals compared to lean controls. However, whereas *Srebp1c* expression was significantly increased in obese unfed animals compared to lean controls, the *Srebp1c* target genes including *Fasn*, *Aacs*, *Sqle*, and *Ldlr* were not (Fig 7G and S8E Fig). Expression of genes in cluster 7 was increased in obese animals in unfed condition to a level similar to fed lean animals. This demonstrates mechanistic differences of impaired feeding-mediated induction of gene expression in the liver of obese animals. Likewise, three clusters (4, 5, and 6) of impaired feeding-repressed genes could be identified (Fig 7H). Expression of genes in cluster 4 was reduced in unfed obese animals (Fig 7I), resulting in a pronounced impaired feeding response compared to genes in cluster 1 (Fig 7I and S8D Fig). Cluster 4 contains genes such as *Pck1*, *Tat*, and *Klf15*, which we characterized to be regulated by the HPA axis during the circadian feeding response (Fig 6F). In agreement, genes in cluster 4 were significantly enriched for genes regulated by dex and not S961 (Fig 7J) in contrast to genes in cluster 1, 5, and 6. In agreement with reduced corticosterone levels in obese animals, we observed reduced GR occupancy to several GR-binding sites (S9A Fig), likely resulting in the attenuated expression of GR target genes in obese animals. In contrast, expression of genes such as *Insig2* and *Angptl4* in clusters 5 and 6 were increased in fed obese animals compared to fed lean controls, and these clusters of genes were primarily enriched for genes regulated by S961 (Fig 7I and 7J). This indicates that expression of these genes is increased as a result of increased activity of insulin-repressed transcription factors such as FOXO1. However, FOXO1 occupancy of chromatin at several FOXO1-binding sites was suppressed in unfed obese animals compared to lean controls (S9B Fig). This suggests that the increased preprandial insulin level observed in the short-term night-restricted fed obese mice was able to impair FOXO1 binding to chromatin. Thus, under these experimental conditions, FOXO1 is likely not involved in the observed dysregulated postprandial expression of genes such as *Ppara*, *Insig2*, and *Angptl4*, and this also explains why expression of FOXO1 target genes such as *G6pc* and *Igfbp1* was suppressed in unfed obese animals compared to lean controls.

## Discussion

Hepatic circadian gene transcription is coupled with environmental cues such as physical activity, light sensed by the suprachiasmatic nucleus (SCN), and feeding behavior. Here, we demonstrate that a large fraction of the circadian transcriptome in the liver is regulated by feeding, and we identified thousands of DNase accessible regions differentially acetylated at H3K27 in response to feeding at the light/dark transition. Importantly, feeding-regulated transcription correlated with differential H3K27Ac DNase-accessible regions. Moreover, we could divide regulatory regions into two major groups: one group regulated by feeding and one group controlled independent of feeding. Both groups had a similar overall circadian H3K27Ac profile. Extensive DNA motif analysis of feeding-regulated enhancers as well as diurnal regulated enhancers uncoupled from feeding uncovered putative transcription factor networks operating at the transition between the unfed and fed state of a circadian rhythm.

In this study, we functionally addressed postprandial repression of gene expression and H3K27 acetylation. We found enrichment of GR, FOXO1, and CREB DNA–binding motifs specifically within DHSs associated with feeding-repressed H3K27Ac and observed that GR and FOXO1 occupancy of chromatin was significantly suppressed by feeding. In addition, the GR and FOXO1 cistromic analysis suggested considerable coregulation of enhancer activity and gene expression by upstream signaling pathways regulating GR and FOXO1 activity. The surge of circulating insulin observed after feeding leads to protein kinase B (PKB/AKT)-dependent FOXO1 phosphorylation and subsequent nuclear exclusion [38] in agreement with the observed reduced postprandial FOXO1 occupancy of chromatin. In parallel, we observed reduced corticosterone levels after feeding in an insulin-independent manner. GR occupancy of chromatin is highly dependent on the level of circulating corticosterone [39]. Accordingly, preprandial injection of dex resulted in recruitment of GR to chromatin in the fed state, suggesting that the level of circulating glucocorticoids is a major determinant for postprandial suppression of GR occupancy of chromatin. Interestingly, despite a genome-wide reappearance of GR occupancy upon preprandial dex injection, only a subset of GR-bound feeding-regulated enhancers was reacetylated at H3K27. Accordingly, only a subset of the feeding-repressed genes was induced by dex, suggesting that GR controls a group of postprandial repressed genes by selective enhancer regulation. Enhancers responsive to dynamic glucocorticoid regulations were highly enriched for canonical GREs and relatively high levels of GR occupancy, suggesting that direct interaction of GR with its canonical binding site drives histone acetylation of specific enhancers. In contrast, other enhancers occupied by GR via degenerated GREs are less likely to be regulated by dynamic glucocorticoid signaling. Thus, apart from being a synchronizer of the intrinsic circadian clock in the liver [40,41], GR also directly regulates expression of a number of circadian-expressed genes controlled by daily fed/fasting rhythms.

Injection of an insulin receptor antagonist identified postprandial repressed genes controlled predominantly by insulin receptor signaling. Interestingly, several of these insulin-regulated genes were not affected by dynamic glucocorticoid signaling, suggesting that postprandial suppression of circulating corticosterone and increased insulin levels independently regulate the mRNA levels of a subset of feeding-regulated genes in the liver. This includes genes such as *Pck1*, *Tat*, *Insig2*, and *G6pc*, regulated primarily by glucocorticoids (*Pck1* and *Tat*) or insulin receptor signaling (*G6pc* and *Insig2*). Yet studies using primary hepatocytes and/or conditional KO models have shown that *Pck1* expression can be regulated by insulin receptor signaling, and *G6pc* and *Insig2* expression can be regulated by GR [42–44]. Thus, we cannot rule out that under certain experimental conditions, genes regulated primarily by glucocorticoids are also under the control of insulin signaling and vice versa. Additionally, increased levels of NEFA in response to insulin resistance may activate transcription factors such as PPARα in the liver [45], which could also contribute to the regulation of genes such as *G6pc*, *Angplt4*, and *Insig2* [46].

When postprandial insulin and glucocorticoid signaling were independently disrupted, we observed partial disruption of postprandial repression of gene expression; however, combined disruption of these signaling pathways resulted in a postprandial expression profile approximating preprandial gene expression. This demonstrates cooperative actions of insulin and corticosterone signaling to repress postprandial gene expression in the liver and emphasizes that signals operating independent of insulin receptor signaling are involved in postprandial repression of transcription in the liver. These signaling pathways may be involved in hepatic metabolic homeostasis of animals lacking both FOXO1 and upstream components suppressing FOXO1 activity such as AKT, IRS, or IR [35,42,47,48]. For example, FGF15/19 has been shown to reduce circulating corticosterone levels [49] and may act in parallel with insulin receptor signaling to regulate postprandial hepatic gene expression [50]. FGF15/19 is secreted from the small intestine in response to feeding, and signaling via the FGFR4/betaKlotho receptor is important for increased postprandial hepatic glycogen storage, protein synthesis, and decreased gluconeogenesis [51,52]. Transcriptional repression of gluconeogenic genes by FGF15/19 involves suppression of CREB, FOXO1, and PPARG Coactivator 1 (PGC-1) activity [51,53]; however, it has yet to be determined whether other transcription factors, such as GR, are involved. Leptin is another possible postprandial suppressor of circulating corticosterone [54]; however, leptin secretion from adipose tissue is dependent on insulin [55], suggesting that leptin is not involved in the glucocorticoid-specific regulation of postprandial gene expression in the liver, independent of insulin signaling.

Circadian gene expression in the liver is altered in diet-induced obese animals, partly linked to perturbed feeding patterns [12–14]. To study the feeding response in diet-induced obese animals, we replicated the experimental feeding setup in lean and corresponding diet-induced obese mice. Using this approach, we observed a clear perturbed feeding response in obese animals. For example, we observed suppressed postprandial lipogenic gene expression (e.g., *Fasn* and *Aacs*) and disrupted preprandial gluconeogenic gene expression (e.g., *G6pc* and *Pck1*. Interestingly, however, the disrupted feeding response in this experimental setup contradicts the general notion that diet-induced hyperinsulimia and insulin resistance leads to increased expression of lipogenenic and gluconeogenic genes by unresolved mechanisms [56]. The discrepancy between this study and other studies may be linked to the short-term night-restricted experimental setup used, which could increase insulin sensitivity. Hence, the preprandial hyperinsulinemia could lead to activation of insulin signaling, abrogating FOXO1 activity. Alternatively, as diet-induced obesity has been shown to dramatically change the circadian gene expression profile [14], the inconsistency may be linked to isolation of livers at different time points during the day.

Consistent with other studies, we observed increased preprandial *Srebp1c* expression in diet-induced obese animals in agreement with hyperinsulimia, yet expression of SREBP1c targets were not induced in the preprandial state nor in response to feeding. This discrepancy may be a result of high pre- and postprandial expression of *Insig2*, a suppressor of SREBP1c activity [57]. Because FOXO1 occupy a number of regions near the *Insig2* gene, elevated *Insig2* expression may be a result of hepatic insulin resistance and increased postprandial FOXO1 activity. However, we observed that FOXO1 occupancy of chromatin is disrupted in diet-induced obese animals in a night-restricted feeding setup, suggesting that *Insig2* expression is not regulated by FOXO1 under these conditions. Instead, *Insig2* may be controlled by increased expression of *Ppara* [46], found to be up-regulated in the fed diet-induced obese animals compared to controls. The observed suppressed FOXO1 occupancy of chromatin in diet-induced obese animals contradicts a number of reports showing increased FOXO1 activity in hyperinsulimic and insulin resistant diet-induced obese animals evaluated by, for example, nuclear abundance [58] and state of phosphorylation [59]. However, as mentioned above, we cannot rule out that the short-term night-restricted feeding regime used in this study increased insulin sensitivity of the liver in diet-induced obese animals, enabling Akt dependent FOXO1 phosphorylation by elevated insulin levels in the preprandial state. In line with this, we observed that postprandial repression of well-known FOXO1 target genes (e.g., *G6pc* and *Igfbp1*) was blunted as a result of reduced preprandial expression.

Unbiased clustering uncovered a group of feeding-regulated genes suppressed in the preprandial state of diet-induced obese animals. This group of genes was particularly enriched for glucocorticoid-sensitive genes. This correlated with reduced preprandial corticosterone levels in obese animals. Dampened circadian corticosterone levels have been reported in diet-induced obese mice [12] and obese humans [60], although other reports have indicated a positive correlation between obesity and corticosterone/cortisol levels [61]. Interestingly, obese, hyperglycemic leptin deficient (*ob/ob*) mice have elevated circadian corticosterone levels [62], demonstrating a clear difference in the activity of the HPA axis between *ob/ob* mice and diet-induced obese C57BL/6 mice. Yet *ob/ob* mice have reduced 11beta-hydroxysteroid dehydrogenase (11β-HSD) activity, likely leading to reduced production of active corticosterone in the liver [63], suggesting that both of these models of obesity may have impaired preprandial GR activity. A body of evidence clearly shows that treatment with glucocorticoids or hypersecretion of cortisol lead to obesity, insulin resistance, and type II diabetes [64], demonstrating that elevated glucocorticoids induce metabolic dysregulation. But clearly metabolic dysregulation caused by diet-induced obesity alone does not necessarily lead to hypercortisolemia. Thus, suppressed preprandial corticosterone levels observed in diet-induced obese animals may be a compensatory mechanism to, for example, impede hyperglycemia when the total energy balance of the animal is in surplus.

Collectively, this study identified hepatic circadian–regulated genes and enhancers controlled by feeding. Specifically, we show that two specific signaling pathways control postprandial repression of hepatic gene expression. On one hand, corticosterone levels decrease in response to food intake, leading to reduced expression of specific genes. This is governed by selective regulation of H3K27Ac by GR, mediated by GR recruitment to canonical GREs. In parallel, food intake leads to increased insulin levels, resulting in reduced expression of a set of glucocorticoid independent genes. Both pathways are disrupted during a feeding response in diet-induced obese animals, and impacting postprandial gene regulation in the liver. Thus, a major part of the hepatic circadian–regulated gene program, controlled by feeding, is operated by combined dynamic glucocorticoid and insulin signaling.

## Materials and methods

### Ethics statement

All mouse work was approved by the Danish Animal Inspectorate (case number 2014−15−0201−00437 and 2017-15-0201-01232), the Danish Environmental Protection Agency, and the Children’s Hospital Boston Institutional Animal Care and Use Committee.

### Animal experiments

#### Experimental setup

Ten-week old C57BL/6N (Taconic Biosciences) male mice were maintained in 12-hour light–dark cycle with lights on at 6 AM (ZT0) and lights off at 6 PM (ZT12), with ad libitum access to chow (Altromin 1324) and water. Four days prior to the experiments, mice were single caged and trained to NRF for which chow was present during the dark period only. On the day of the experiment, one group of mice was euthanized at ZT10, one group of mice was presented with food at ZT12 and was euthanized at ZT14 (ZT14-fed), and one group of mice remained unfed and was euthanized at ZT14 (ZT14-unfed). All mice were euthanized by cervical dislocation. Livers were isolated and frozen in liquid nitrogen.

#### Injection of dex and S961

Ten-week old C57BL/6N were subjected to night-restricted feeding as described above. On the day of the experiment, vehicle (PBS), water-soluble dex (1 mg/kg in PBS) (Sigma D2915), and/or S961 (44 nmol/kg in PBS) (generous gift from Novo Nordisk) were injected intraperitoneal (i.p.) 30 minutes before ZT12. Livers were isolated from unfed and fed mice at ZT14.

#### L-IRSdKO experiments

L-IRSdKO (C57BL/6J background) mice were generated as previously described [35] and subjected to the NRF routine at 10 weeks of age. On the day of the experiment, dex was injected i.p. 30 minutes before ZT12, and food was presented at ZT12. Livers were isolated from fed mice at ZT14.

#### L-GRKO experiments

A liver-specific adeno-associated virus (AAV) containing CRE recombinase driven by the liver-specific Tbg promoter (AAV8.TBG.PI.Cre.rBG, Lot #CS1129, Penn virus core facility) or AAV containing GFP driven by Tbg (AAV8.TBG.PI.eGFP.WPRE.bGH, Lot #V6085R, Penn virus core facility) as control was diluted in sterile PBS and delivered to mice at a titer of 10^11^ GC per mouse. Specifically, 100 μl AAV diluted in PBS was injected in the tail vein using 23-G needle into homozygote adult GR^fl/fl^ mice (Nr3c1tm2Gsc [65] on a C57BL/6N background) 12 days before the NRF routine. Livers were isolated from unfed mice at ZT14.

#### HFD experiments

Five-week old male mice were subjected to HFD (D12492 Research Diets, 60% energy from fat) for 10 or 20 weeks and were then subjected to the NRF routine. Livers were isolated from unfed and fed mice at ZT14.

#### Glucose tolerance test

IPGTT was performed by i.p. D-glucose (Sigma G8270) injection at 2 g/kg to mice fasted for approximately 5 hours and measuring blood glucose at 0, 5, 10, 20, 60, and 120 minutes from injection time. The blood glucose was measured with a glucometer (FreeStyle Freedom Lite) from a tail puncture with a needle.

### Metabolic assays

Approximately 300 μl of blood was collected postmortem and stored on ice for further analysis. The blood was centrifuged at 10,000 rcf for 10 minutes at 4°C and prepared for insulin, corticosterone, and NEFA measurements, as indicated by UltraSensitive Mouse Insulin ELISA kit (Crystal Chem), the Corticosterone ELISA kit (Enzo Life Sciences), and the NEFA quantification kit using the ACS-ACOD method (Wako #434–91795, #436–91995, #270–77000) according to manufacturer instructions. Liver triglyceride levels were measured as described previously [66].

### RNA extraction and mRNA quantitation by RT-qPCR

Approximately 5 mg of liver tissue was homogenized using Ultra-Turrax, and RNA was purified using TRIzol-RNA lysis reagent (Thermo Fisher) in EconoSpin columns (Epoc Life) according to manufacturer instructions. The amount of 1 μg of total RNA was exposed to 10 U DNAse I (Thermo Fisher) for 15 minutes at 37°C, followed by cDNA synthesis using random primers and Moloney murine leukemia virus reverse transcriptase (Thermo Fisher). Expression levels were measured by qPCR using SYBR Green reagent mix (Roche 06924204001) and primers listed in S2 Table. The qPCR was performed on a LightCycler480 (Roche). The expression was normalized to TFIIB expression.

### Immunoblotting

Approximately 20 mg of liver was shortly homogenized in 700 μl lysis buffer (50 mM Tris-HCl [pH 6.8], 10% glycerol, 2.5% SDS, 10 mM beta-glycerolphosphate, 10 mM NaF, 0.1 mM NaOrthovanadate, 1 mM PMSF, and 1x protease inhibitor cocktail) and treated with benzonase (Sigma E1014). Protein concentration was determined using the Pierce BCA protein assay kit (Thermo Fisher 23225). A total of 80 μg protein was mixed with laemmli buffer and separated by SDS-PAGE and blotted onto a PVDF membrane (10600023, Amersham Hybond P0.45 PVDF, lot#G9889898). The membrane was probed with primary antibody against GR (sc-1004, Santa Cruz), FOXO1 (sc-11350, Santa Cruz), TFIIB (sc-225, Santa Cruz) or β−Tubulin (05–661 Merck Millipore), and secondary HRP conjugated antibody (DAKO).

### ChIP

In short, ChIP was performed as described previously [66] with minor modifications. Frozen livers (approximately 50 mg per IP) were homogenized in 1% Formaldehyde PBS solution using Ultra-Turrax homogenizer on level 5 for 10 seconds, with following cross-linking at room temperature for 10 minutes. The suspension was quenched by adding 0.125 M glycine and incubating additional 10 minutes at room temperature. Cross-linked cells were washed in PBS, resuspended in lysis buffer (0.1% SDS, 1% Triton X-100, 0.15 M NaCl, 1 mM EDTA, 20 mM TrisHCl [pH 8.0], and BSA 1 mg/ml), and sonicated using Bioruptor (Diagenode) or ME220 Focused-ultrasonicator (Covaris). Chromatin was immunoprecipitated over night at 4 °C using antibodies and Protein A/G agarose beads (Santa Cruz, sc-2003). H3K27Ac ChIP was done with 0.2 μl/IP of H3K27Ac antibody Ab4729 (Abcam). GR ChIP antibody cocktail consisted of 1 μg/IP of MA1-510 (Thermo Fisher), 1 μg/IP of PA1-511a (Thermo Fisher), and 1μg/IP of sc-1004 (Santa Cruz). FOXO1 ChIP was done with 3 μg/IP of sc-11350 (Santa Cruz). Immunocomplexes were washed, and chromatin was eluted (1% SDS with 0.1 M NaHCO_3_) and decross-linked over night at 65 °C. DNA was phenol/chloroform purified and ethanol precipitated. Recovery was analyzed by qPCR using primers listed in S2 Table and/or sequenced.

### DNase digestion of chromatin

Livers were isolated from 3–4 mice in each treatment group (ZT14-fed or ZT14-unfed), and nuclei were immediately purified from a pool of 200–300 mg liver tissue using a previously described protocol [24]. This was repeated to form a second biological replicate. Purified nuclei were resuspended in buffer (15 mM Tris-HCl [pH 8.0], 15 mM NaCl, 60 mM KCl, 1 mM EDTA, 0.5 mM EGTA, 0.5 mM Spermidine, and protease inhibitors) in a final concentration of 10 million nuclei per milliliter. DNase digestions were performed by adding 100 μl 10X digestion buffer (60 mM CaCl_2_ and 750 mM NaCl) containing 40 U, 60 U, or 80 U of DNase I (Sigma). Digestions were incubated for 3 minutes at 37 °C, and reactions were terminated by addition of 1 volume of stop buffer (50 mM Tris-HCl, 100 mM NaCl, 0.1% SDS, 100 mM EDTA and 50 μg/ml Proteinase K [Ambion]). Digested chromatin was incubated at 55 °C for 2 hours and stored at 4 °C until further use. DNase I digestion efficiency was evaluated by qPCR, and samples with optimal digestion efficiency were incubated with 90 μg/ml RNase A (Sigma) for 30 minutes at 37 °C before 50- to 500-bp DNA fragments were purified using ultracentrifugation. DNA was subsequently phenol/chloroform purified and ethanol precipitated. Samples treated with 60 U and 80 U of DNase I were sequenced.

### Illumina sequencing

#### RNA-seq

RNA quality was assessed using the Fragment Analyzer (AATI). Total RNA (1 μg) was prepared for sequencing using polydT enrichment or ribosome depletion according to manufacturer (Illumina) instructions. Subsequent library preparation was performed using the NEBNext RNA library prep kit for Illumina. Library quality was assessed using the Fragment Analyzer followed by library quantification (Illumina library quantification kit). Sequencing was carried out on a HiSeq1500 platform (Illumina).

#### ChIP-seq and DNase-seq

ChIP-seq and DNase-seq libraries were constructed essentially as described previously [66] using the NEBNext DNA library prep kit according to manufacturer (NEB) instructions. Sequencing was carried out on a HiSeq1500 platform (Illumina). Sequenced DNA was aligned to the mouse genome assembly, mm9, using STAR [67]. See S1 Table for details.

### Sequencing analysis

#### RNA-seq

Sequenced reads were normalized to RPKM (RNA-seq), and differentially regulated genes were identified using DESeq2 with *n* = 2–4 [68]. Heatmaps were generated from log2 fold change expression or z-score–normalized RPKM values (z = [x-mean]/standard deviation) and visualized using R (pheatmap package) on selected sets of genes. The average z-score for a group of genes was calculated as the mean of z-scores for a given treatment across a given set of genes. Principal component analysis was performed using R (prcomp package). Hierarchical clustering was performed using the Ward method as a part of the pheatmap package. GO analysis was performed by GOseq [69].

#### DNase-seq

Sequenced tags were normalized to 10 million reads using HOMER [27]. DHSs were identified based on combined reads from all DNase replicates using HOMER at FDR rate threshold = 0.001 and tag threshold of 25. Quantification of DHS in fed and unfed condition was performed using pooled sequencing data from 60 U and 80 U DNase treatments from each biological replicate.

#### ChIP-seq

Sequenced tags were normalized to 10 million reads using HOMER [27]. Quantification of H3K27Ac at DHS was performed using an 800-bp window relative to the center of the DHS. Differentially H3K27Ac was identified using DESeq2 [68]. Heatmaps illustrating H3K27Ac were generated from z-score–normalized read counts using R (pheatmap package), and hierarchical clustering was performed using the Ward method as a part of the pheatmap package. GR and FOXO1 peaks were identified from replicate ChIP-seq using HOMER at FDR rate threshold = 0.001 and tag threshold of 25 using input chromatin controls. Overlapping replicate concordant GR and FOXO1 peaks were identified between two replicates using BEDtools. Enrichment of differentially regulated H3K27Ac as well as GR, FOXO1, and CREB occupancy near differentially regulated genes was analyzed using BEDtools, and sets of randomly selected genes were used to calculate the relative enrichment/frequency. Genes were randomly selected from all annotated refseq genes in the mm9 genome assembly. Heatmaps illustrating FOXO1 and GR occupancy were generated using MeV [70].

### Motif analysis

De novo motif analysis, log odds motif score, and enriched motifs were identified using HOMER [27]. A set of 2,000 randomly picked DHSs from the total amount of DHSs identified in liver was used as background for the motif enrichment analysis. The GC content of the randomly selected DHSs was 48%, while the mean GC content of DHSs in clusters 1–4 (Fig 3A) ranged from 46% to 49%. Motif analysis using IMAGE was performed using quantified H3K27Ac ChIP-seq reads at DHSs and RNA-seq reads at genes as described previously [26]. Three biological H3K27Ac ChIP-seq replicates were used from livers of mice euthanized at ZT10, ZT14-fed, and ZT14-unfed. Gene expression input for IMAGE was based on replicate RNA-seq data from ZT10, ZT14-fed, and ZT14-unfed.

### Statistical analysis

Statistical analysis was performed using Student *t* test, Mann–Whitney–Wilcoxon test, and Kolmogorov–Smirnov test, as indicated in figure legends. FDR for NGS-read counts at genes, DHSs, and ChIP-seq peaks between different conditions were calculated using DESeq2 [68]. Enrichment of DNA motifs was calculated by HOMER [27] and IMAGE [26]. GO enrichment was performed by GOseq [69].

### Next-generation sequencing data

All sequencing data can be found at GEO: GSE119713. Previously published sequencing data used for analysis include the following: Circadian RNA-seq: GSE73554 [1]; Circadian H3K27Ac ChIP-seq: GSE60430 [16]; and CREB ChIP-seq: GSE45674 [31] and GSE72084 [30].

## Supporting information

S1 Fig Correlation of replicate DNase-seq experiments in livers isolated from fed and unfed animals at ZT14. Sequenced tags from DNase-seq libraries generated from 60 U and 80 U DNase I digestions of nuclei isolated from mouse liver tissue are quantified at the 83,592 identified DHSs. Red line represents the linear regression, and Pearson correlation coefficient is indicated for pairwise correlation of replicates (r1 and r2). Numerical values are available in S8 Data. DHS, DNase hypersensitive site; ZT, Zeitgeber time.
(TIF)

S2 Fig Correlation of replicate H3K27Ac ChIP-seq experiments in livers isolated from unfed and fed animals at ZT10 and ZT14. Sequenced tags from H3K27Ac ChIP-seq libraries generated from mouse liver tissue are quantified at 1,000 bp surrounding the 83,592 identified DHSs. DHSs with less than 20 H3K27Ac ChIP-seq tags are excluded from the analysis. Red line represents the linear regression, and Pearson correlation coefficient is indicated for pairwise correlation of replicates. (A) Correlation between three biological replicates (r1–r3) at ZT10. (B) Correlation between three biological replicates (r1–r3) at ZT14-unfed. (C) Correlation between three biological replicates (r1–r3) at ZT14-fed. Numerical values are available in S9 Data. ChIP, chromatin immunoprecipitation; DHS, DNase hypersensitive site; H3K27Ac, histone 3 lysine 27 acetylation; ZT, Zeitgeber time.
(TIF)

S3 Fig De novo motif analysis of DHSs associated with feeding-regulated H3K27Ac. De novo motifs analysis was performed using HOMER on 1,437 and 1,896 DHSs associated with feeding-induced and feeding-repressed H3K27Ac, respectively. The five most enriched motifs are shown together with the frequency of the motifs within the DHSs (percent target) and the transcription factors likely to interact with the motifs. DHS, DNase hypersensitive site; H3K27Ac, histone 3 lysine 27 acetylation; HOMER, hypergeometric optimization of motif enrichment.
(TIF)

S4 Fig GR and FOXO1 occupancy of feeding-regulated enhancers. (A) De novo motif analysis of regions occupied by GR and FOXO1 (ZT14-unfed). The three most enriched motifs are displayed. (B) Evaluation of FOXO1 antibody by western blotting using liver protein extracts from WT and FOXO1 KO animals. (C) Co-occupancy of GR, FOXO1, and CREB using CREB-binding sites from GSE72084. (D) Frequency of Rep. genes with at least one GR, FOXO1, or CREB peak within 50 kb of the TSS. Frequency is relative to GR, FOXO1, and CREB binding within 50 kb of TSS of sets of 300 randomly selected genes. Data is presented as mean ± SEM (*n* = 6). (E) Number of GR, FOXO1, and CREB ChIP-seq peaks located within 50 kb of TSS of Ind. and Rep. genes. The number of GR, FOXO1, and CREB ChIP-seq peaks within 50 kb of TSSs of 200 randomly selected genes is included as control. Statistical significance is calculated using a Mann–Whitney–Wilcoxon Test. (F) Tag density of GR, FOXO1, and CREB occupancy at DHS within 50 kb of TSS of Ind. genes and Rep. genes. Statistical significance is calculated using a Mann–Whitney–Wilcoxon Test. Numerical values for panels C–F are available in S10 Data. ChIP, chromatin immunoprecipitation; CREB, cAMP responsive element binding protein; FOXO1, Forkhead box O1; GR, glucocorticoid receptor; Ind. gene, feeding-induced gene; KO, knockout; Rep. gene, feeding-repressed gene; TSS, transcription start site; WT, wild-type; ZT, Zeitgeber time.
(TIF)

S5 Fig Correlation of dex-regulated H3K27Ac with feeding-regulated H3K27Ac and GR, CREB and FOXO1 occupancy at GR-occupied DHSs associated with feeding–down-regulated H3K27Ac. (A) Correlation of dex-regulated H3K27Ac with feeding-regulated H3K27Ac at GR-binding sites. (B) Correlation of dex-regulated H3K27Ac with feeding-regulated GR occupancy at GR-binding sites. (C) Correlation of dex-regulated H3K27Ac with the level of GR occupancy in unfed condition. (D) Correlation of dex-regulated H3K27Ac with the GRE motif score. The degree of correlation between each of the two variables in panels A–D was tested by a Pearson correlation (r) and the red line represents a linear regression. (E) ROC analysis of GR, FOXO1, and CREB occupancy together with GR, FOXO1, and CREB motif scores within DHSs associated with dex-regulated H3K27Ac. Numerical values are available in S11 Data. CREB, cAMP responsive element binding protein; dex, dexamethasone; DHS, DNase hypersensitive site; FOXO1, Forkhead box O1; GR, glucocorticoid receptor; GRE; H3K27Ac; ROC, receiver operating characteristic.
(TIF)

S6 Fig Regulation of H3K27Ac and mRNA levels by dex. (A) FOXO and CREB (B) occupancy of DHS acetylated at H3K27 in response to dex treatment. Differential H3K27Ac in response to dex is indicated with green color variance in the figure. Statistical analysis is performed by a Mann–Whitney–Wilcoxon test. (C) RT-qPCR analysis of gene expression in the livers of mice injected with vehicle (PBS), dex, or S961. Livers were isolated from unfed or fed mice at ZT14. Data are presented as mean ± SEM (*n* = 8–12). Statistical significance compared to fed-veh is calculated using Student *t* test. ***p < 0.001. (D–G) Examples of H3K27Ac and GR occupancy near genes regulated by dex or S961 injection. Numerical values for panels A–C are available in S12 Data. Bed graphs in panels C–G are available at GEO: GSE119713. dex, dexamethasone; DHS, DNase hypersensitive site; GR, glucocorticoid receptor; H3K27Ac, histone 3 lysine 27 acetylation; RT-qPCR, reverse transcription quantitative PCR; ZT, Zietgeber time.
(TIF)

S7 Fig Hepatic gene expression in animals with disrupted glucocorticoid and insulin receptor signaling. (A) Experimental setup of S961 injection and subsequent isolation of livers at ZT14. (B) Experimental setup of AAV–GFP and AAV–CRE injection of GR^fl/fl^ mice. (C) RT-qPCR analysis of GFP, CRE, and GR expression two weeks post AAV injection. Data are presented as mean ± SEM (*n* = 4). (D) Western blot analysis of GR expression. (E) Quantification of mRNA expression (RNA-seq) in livers from GR^fl/fl^ mice injected with AAV–GFP and AAV–CRE. Feeding-repressed genes regulated by dex, S961, or dex + S961 were analyzed separately. Log2 RPKM is visualized from four biological replicates (R1–R4). Statistical significance is calculated using a Mann–Whitney–Wilcoxon test of the mean of four biological replicates. (F) Quantification of mRNA expression (RNA-seq) in livers from WT and L-IRSdKO. Feeding-repressed genes regulated by dex, S961, or dex + S961 were analyzed separately. Log2 RPKM is visualized from four biological replicates (R1–R4). Statistical significance is calculated using a Mann–Whitney–Wilcoxon test of the mean of four biological replicates. Numerical values for panels B, C, E, and F are available in S13 Data. AAV, adeno-associated virus; CRE, CREB response element; dex, dexamethasone; GFP, Green fluorescent protein; GR, glucocorticoid receptor; L-IRSdKO, liver specific IRS double knockout; RPKM, reads per kilo base per million mapped reads; RT-qPCR, reverse transcription quantitative PCR; WT, wild-type; ZT, Zietgeber time.
(TIF)

S8 Fig Experimental setup for diet-induced obesity and analysis of differential gene expression. (A) Weight gain of diet induced obese animals compared to chow-fed controls. NRF was initiated 10 weeks after the start of HFD feeding. Chow fed (*n* = 16) and HFD fed (*n* = 16). (B) Experimental setup of NRF and indicated endpoints for the experiment. Livers were isolated at ZT14 from fed and unfed animals. (C) Preprandial (ZT14-unfed) blood was extracted from animals trained to night-restricted feeding at 10 and 20 weeks after initiation of HFD feeding. Glucose, insulin, and corticosterone were measured from at least five independent animals. Individual data points are marked in each panel. (D) Gene expression analysis of genes differentially expressed in obese animals. For each cluster, the percentage of differentially induced (cluster 1, 3, 7, and 8) and repressed genes (cluster 1, 4, 5, and 6) at FDR < 0.05 is indicated at the top. (E) Expression of *Srebp1c* and *Srebp1c* target genes (*Aacs*, *Sqle*, and *Ldlr*), measured by RNA-seq (*n* = 3). Data in panels A, C, and E are presented as mean ± SEM. Statistical significance is calculated using Student *t* test. *** indicates *p* < 0.001 or otherwise indicated in figure. Numerical values for panels A and C–E are available in S14 Data. FDR, false discovery rate; HFD, high-fat diet; NRF, night-restricted feeding; ZT, Zeitgeber time.
(TIF)

S9 Fig Hepatic GR and FOXO1 occupancy in fed and unfed lean and diet-induced obese animals. (A) Occupancy of GR at seven different GRBS (1–7) and one negative ctrl. (B) Occupancy of FOXO1 at seven different FOXO-binding sites (FOXO #1–7) and one negative (ctrl). Data are presented as mean ± SEM (*n* = 5–6). Statistical significance is calculated using Student *t* test. Numerical values for panels are available in S15 Data. ctrl, control site; FOXO1, Forkhead box O1; GR, glucocorticoid receptor; GRBS, GR binding site.
(TIF)

S1 Table Summary of Illumina sequencing data. (PDF)

S2 Table Sequence of primers used for qPCR analysis. qPCR, quantitative PCR.
(PDF)

S1 Data Numerical values for Fig 1. Panels 1B, 1C, 1D, 1E, 1F, 1G, 1H, and 1I.
(XLSX)

S2 Data Numerical values for Fig 2. Panels 2A, 2B, 2C, 2D, 2E, 2F, and 2G.
(XLSX)

S3 Data Numerical values for Fig 3. Panels 3A, 3B, 3D, and 3E.
(XLSX)

S4 Data Numerical values for Fig 4. Panels 4A, 4B, 4C, 4D, 4E, 4F, 4G, and 4H.
(XLSX)

S5 Data Numerical values for Fig 5. Panels 5A, 5C, 5D, 5E, 5F, 5G, 5H, 5I, 5J, 5K, 5L, 5M, 5N, and 5O.
(XLSX)

S6 Data Numerical values for Fig 6. Panels 6A, 6B, 6C, 6D, 6E, 6F, 6G, 6I, and 6J.
(XLSX)

S7 Data Numerical values for Fig 7. Panels 7A, 7B, 7C, 7E, 7G, 7H, 7I, and 7J.
(XLSX)

S8 Data Numerical values for S1 Fig. (XLSX)

S9 Data Numerical values for S2 Fig. (XLSX)

S10 Data Numerical values for S4 Fig. Panels S4C, S4D, S4E, and S4F.
(XLSX)

S11 Data Numerical values for S5 Fig. Panels S5A, S5B, S5C, and S5E.
(XLSX)

S12 Data Numerical values for S6 Fig. Panels S6A, S6B, S6C, S6D, S6E, S6F, and S6G.
(XLSX)

S13 Data Numerical values for S7 Fig. Panels S7C, S7E, and S7F.
(XLSX)

S14 Data Numerical values for S8 Fig. Panels S8A, S8C, S8D, and S8E.
(XLSX)

S15 Data Numerical values for S9 Fig. Panels S9A and S9B.
(XLSX)

## Abbreviations

11β-HSD: 11beta-hydroxysteroid dehydrogenase
AAV: adeno-associated virus
AKT: protein kinase B
ANGPTL4: angiopoietin-like 4
BMAL: brain and muscle Arnt-like protein
ChIP-seq: chromatin immunoprecipitation-sequencing
CLOCK: circadian locomotor output cycles kaput
CRE: CREB response element
CREB: cAMP responsive element binding protein
dex: dexamethasone
DHS: DNase hypersensitive site
DR-1: direct repeat one
FASN: fatty acid synthase
FDR: false discovery rate
FOXO1: Forkhead box O1
GCK: glucokinase
GO: gene ontology
GR: glucocorticoid receptor
GRBS: GR-binding site
H3K27A: histone 3 lysine 27 acetylation
HFD: high-fat diet
HOMER: hypergeometric optimization of motif enrichment
HPA: hypothalamic–pituitary–adrenal
HRE: heat shock response element
IMAGE: integrated analysis of motif activity and gene expression
i.p.: intraperitoneal
IRF: interferon regulatory factor
MA: M (log ratio) and A (mean average) scales
NEFA: nonesterified fatty acid
NRF: night-restricted feeding
ob/ob: leptin deficient
PCK1: phosphoenolpyruvate carboxykinase
PGC-1: PPARG Coactivator 1
PKB: protein kinase B
RAR: retinoic acid receptor
REVERB: reverse c-erbA
ROC: receiver operating characteristic
ROR: RAR-related orphan receptor
RORC: RAR-related orphan receptor C
RPKM: reads per kilo base per million mapped reads
RT-qPCR: reverse transcription quantitative PCR
SCN: suprachiasmatic nucleus
TSS: transcription start site
WT: wild-type
ZT: Zeitgeber time
