# Supplementary figures and images for: Insulin signaling and reduced glucocorticoid receptor activity attenuate postprandial gene expression in liver
Source: PLoS Biol. 2018 Dec 10;16(12):e2006249. doi: 10.1371/journal.pbio.2006249 (PMC6301715; doi:10.1371/journal.pbio.2006249)

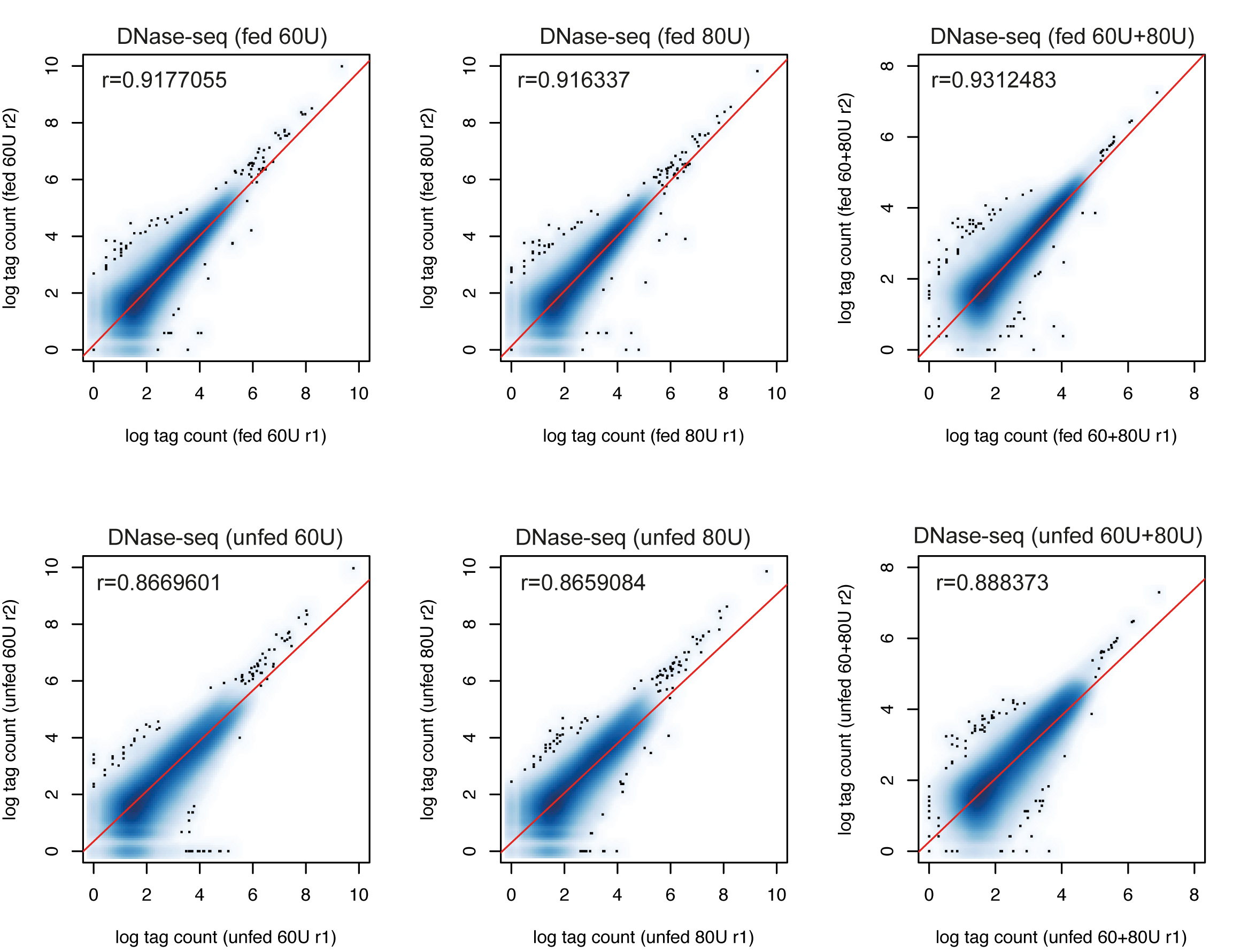

Supplement: S1 Fig — Sequenced tags from DNase-seq libraries generated from 60 U and 80 U DNase I digestions of nuclei isolated from mouse liver tissue are quantified at the 83,592 identified DHSs. Red line represents the linear regression, and Pearson correlation coefficient is indicated for pairwise correlation of replicates (r1 and r2). Numerical values are available in S8 Data. DHS, DNase hypersensitive site; ZT, Zeitgeber time. (TIF) [file pbio.2006249.s001.tif]

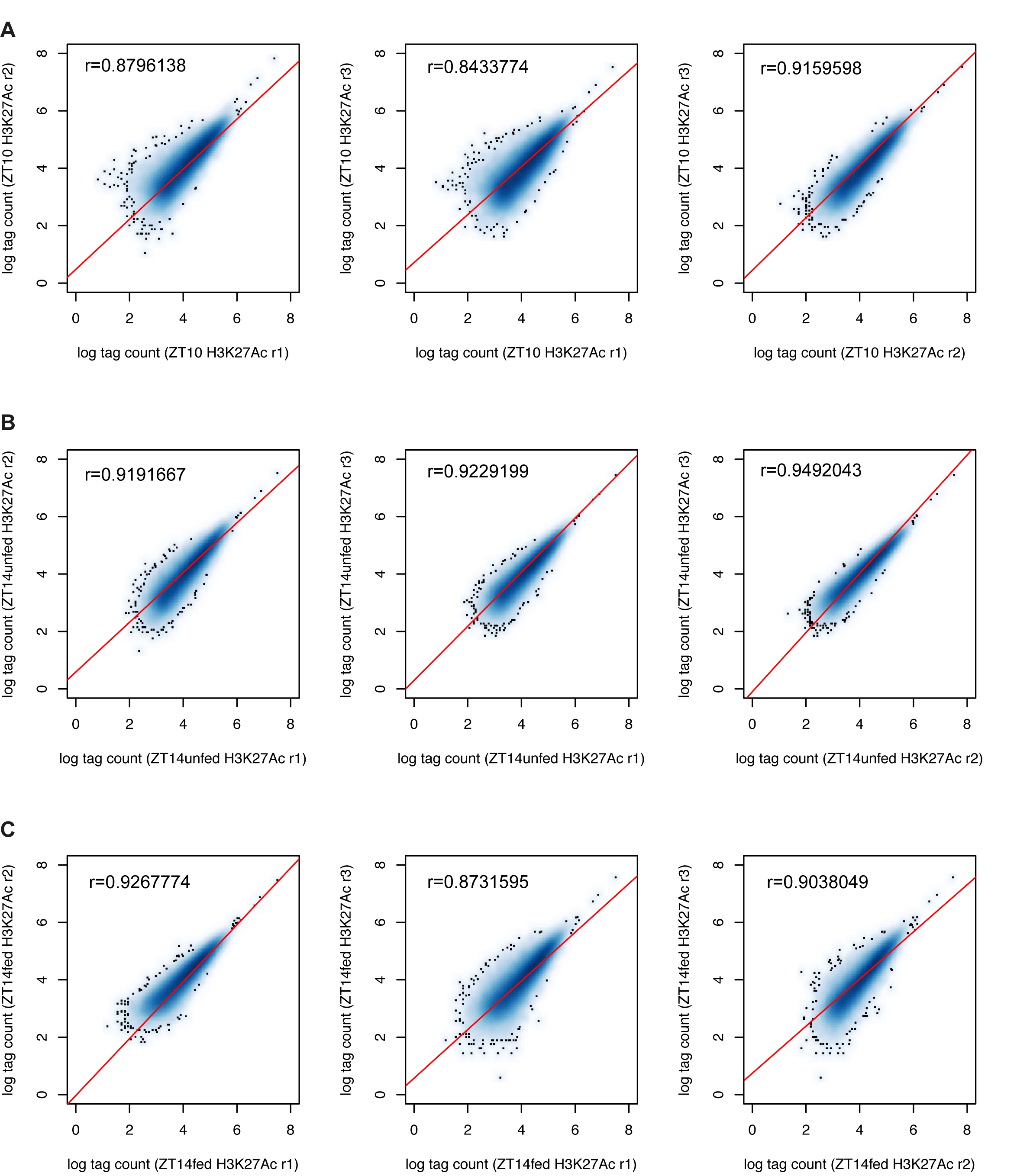

Supplement: S2 Fig — Sequenced tags from H3K27Ac ChIP-seq libraries generated from mouse liver tissue are quantified at 1,000 bp surrounding the 83,592 identified DHSs. DHSs with less than 20 H3K27Ac ChIP-seq tags are excluded from the analysis. Red line represents the linear regression, and Pearson correlation coefficient is indicated for pairwise correlation of replicates. (A) Correlation between three biological replicates (r1–r3) at ZT10. (B) Correlation between three biological replicates (r1–r3) at ZT14-unfed. (C) Correlation between three biological replicates (r1–r3) at ZT14-fed. Numerical values are available in S9 Data. ChIP, chromatin immunoprecipitation; DHS, DNase hypersensitive site; H3K27Ac, histone 3 lysine 27 acetylation; ZT, Zeitgeber time. (TIF) [file pbio.2006249.s002.tif]

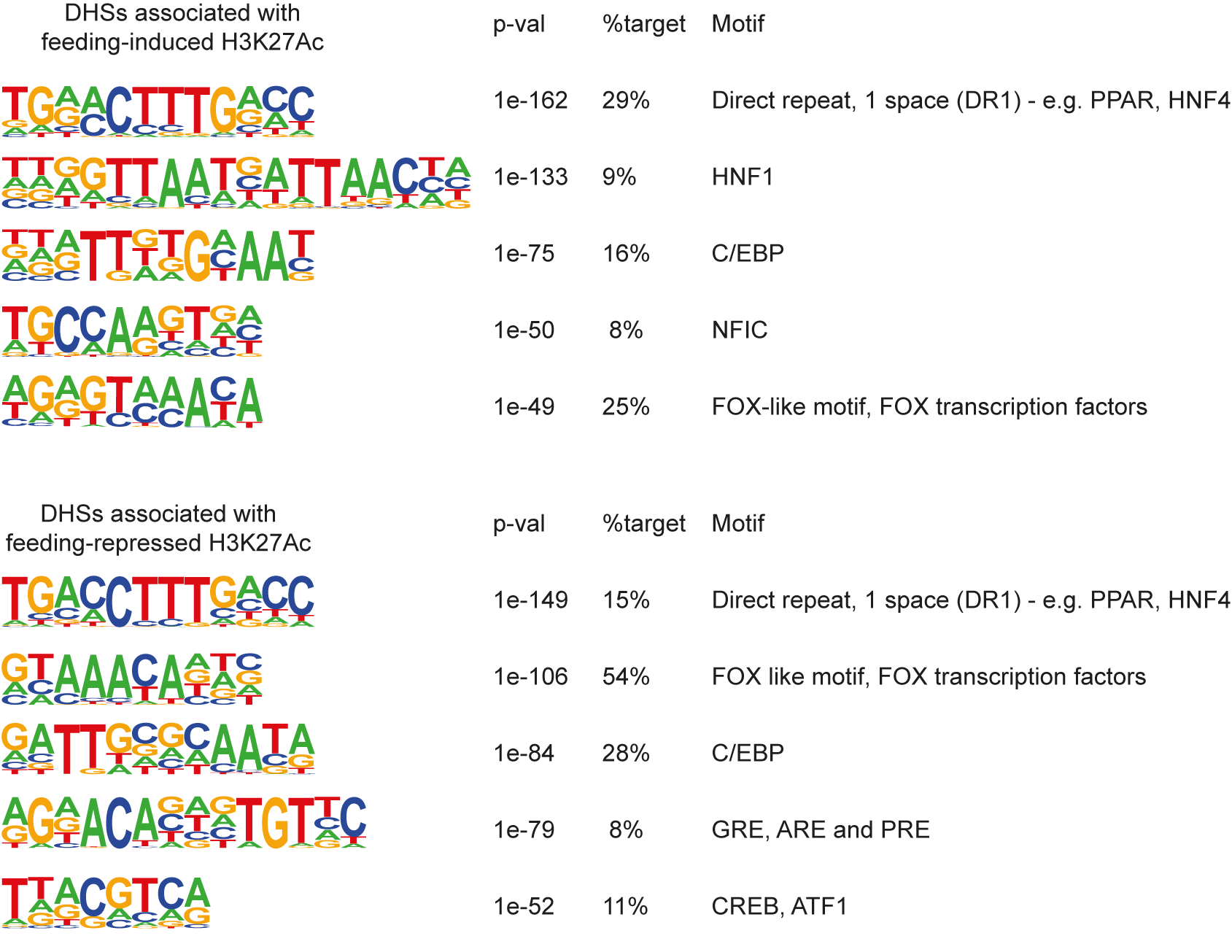

Supplement: S3 Fig — De novo motifs analysis was performed using HOMER on 1,437 and 1,896 DHSs associated with feeding-induced and feeding-repressed H3K27Ac, respectively. The five most enriched motifs are shown together with the frequency of the motifs within the DHSs (percent target) and the transcription factors likely to interact with the motifs. DHS, DNase hypersensitive site; H3K27Ac, histone 3 lysine 27 acetylation; HOMER, hypergeometric optimization of motif enrichment. (TIF) [file pbio.2006249.s003.tif]

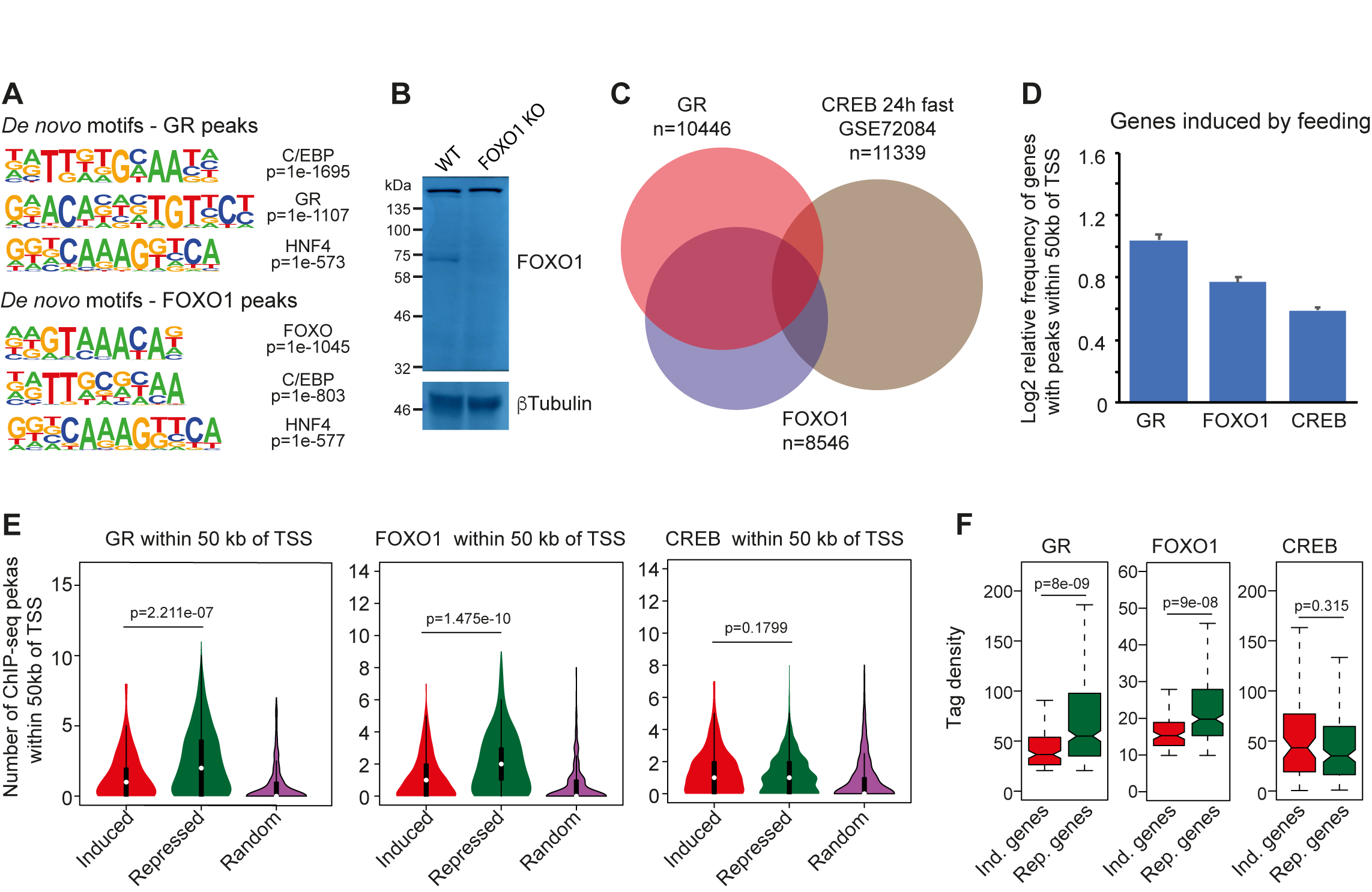

Supplement: S4 Fig — (A) De novo motif analysis of regions occupied by GR and FOXO1 (ZT14-unfed). The three most enriched motifs are displayed. (B) Evaluation of FOXO1 antibody by western blotting using liver protein extracts from WT and FOXO1 KO animals. (C) Co-occupancy of GR, FOXO1, and CREB using CREB-binding sites from GSE72084. (D) Frequency of Rep. genes with at least one GR, FOXO1, or CREB peak within 50 kb of the TSS. Frequency is relative to GR, FOXO1, and CREB binding within 50 kb of TSS of sets of 300 randomly selected genes. Data is presented as mean ± SEM (n = 6). (E) Number of GR, FOXO1, and CREB ChIP-seq peaks located within 50 kb of TSS of Ind. and Rep. genes. The number of GR, FOXO1, and CREB ChIP-seq peaks within 50 kb of TSSs of 200 randomly selected genes is included as control. Statistical significance is calculated using a Mann–Whitney–Wilcoxon Test. (F) Tag density of GR, FOXO1, and CREB occupancy at DHS within 50 kb of TSS of Ind. genes and Rep. genes. Statistical significance is calculated using a Mann–Whitney–Wilcoxon Test. Numerical values for panels C–F are available in S10 Data. ChIP, chromatin immunoprecipitation; CREB, cAMP responsive element binding protein; FOXO1, Forkhead box O1; GR, glucocorticoid receptor; Ind. gene, feeding-induced gene; KO, knockout; Rep. gene, feeding-repressed gene; TSS, transcription start site; WT, wild-type; ZT, Zeitgeber time. (TIF) [file pbio.2006249.s004.tif]

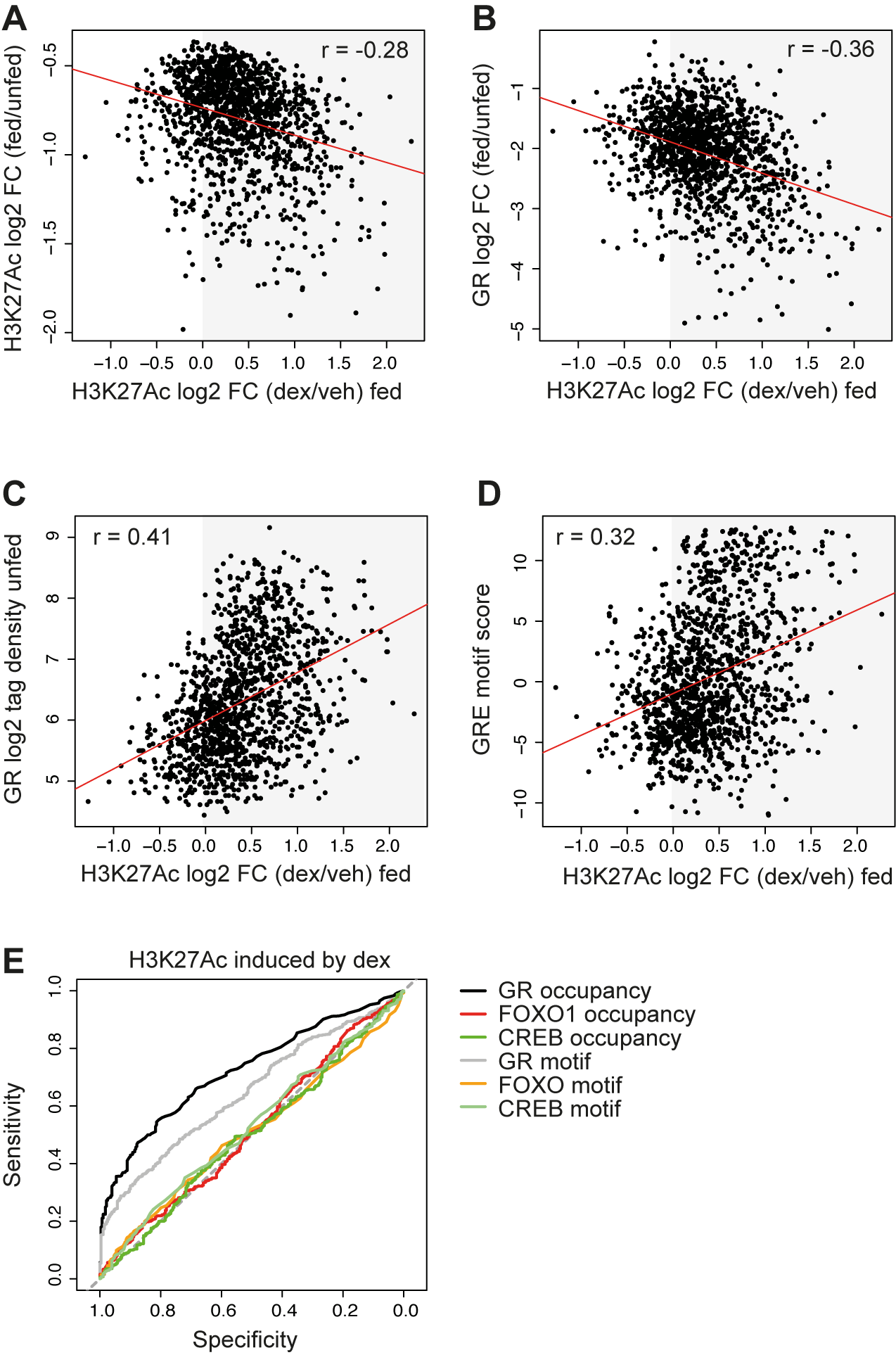

Supplement: S5 Fig — (A) Correlation of dex-regulated H3K27Ac with feeding-regulated H3K27Ac at GR-binding sites. (B) Correlation of dex-regulated H3K27Ac with feeding-regulated GR occupancy at GR-binding sites. (C) Correlation of dex-regulated H3K27Ac with the level of GR occupancy in unfed condition. (D) Correlation of dex-regulated H3K27Ac with the GRE motif score. The degree of correlation between each of the two variables in panels A–D was tested by a Pearson correlation (r) and the red line represents a linear regression. (E) ROC analysis of GR, FOXO1, and CREB occupancy together with GR, FOXO1, and CREB motif scores within DHSs associated with dex-regulated H3K27Ac. Numerical values are available in S11 Data. CREB, cAMP responsive element binding protein; dex, dexamethasone; DHS, DNase hypersensitive site; FOXO1, Forkhead box O1; GR, glucocorticoid receptor; GRE; H3K27Ac; ROC, receiver operating characteristic. (TIF) [file pbio.2006249.s005.tif]

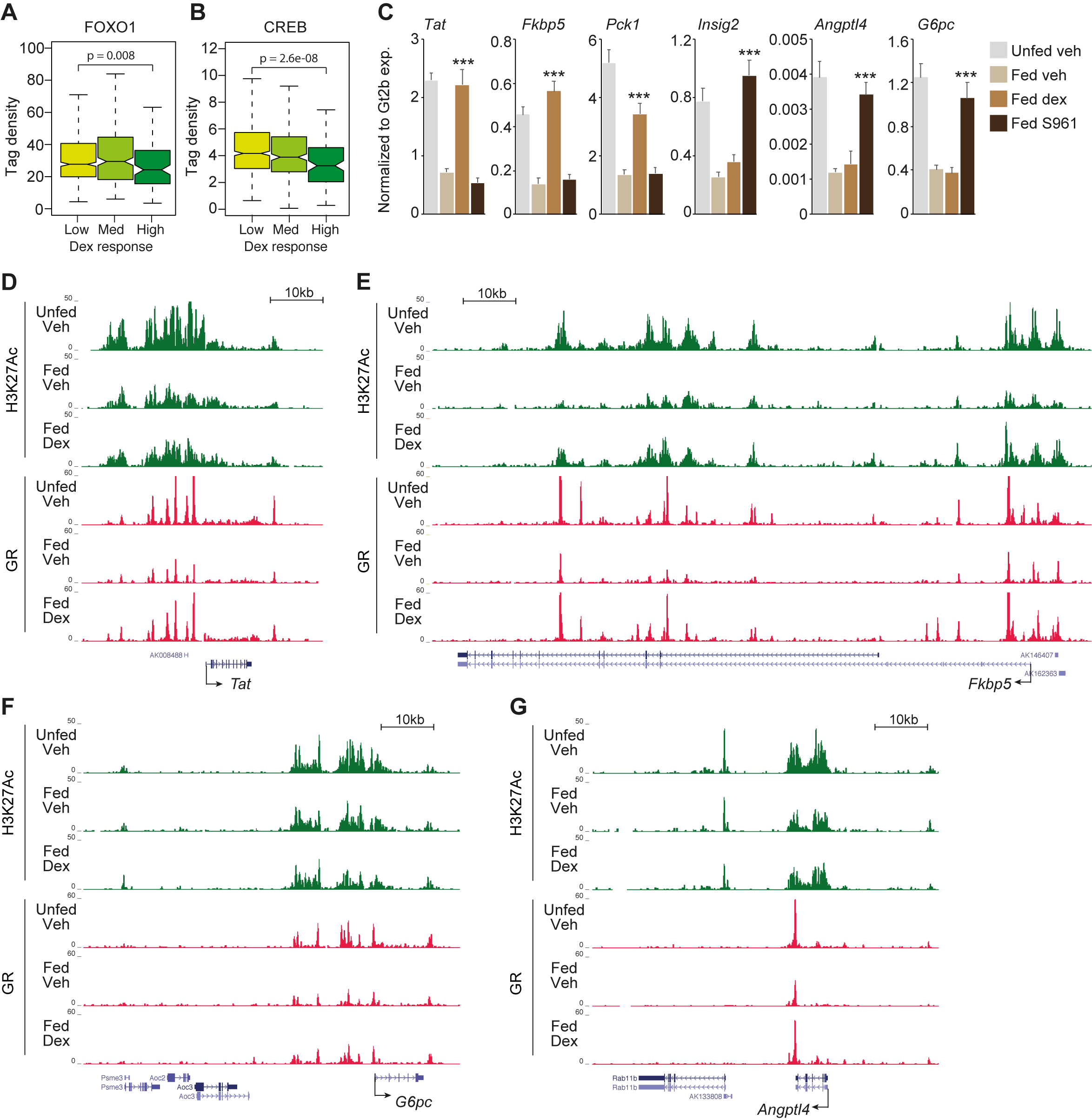

Supplement: S6 Fig — (A) FOXO and CREB (B) occupancy of DHS acetylated at H3K27 in response to dex treatment. Differential H3K27Ac in response to dex is indicated with green color variance in the figure. Statistical analysis is performed by a Mann–Whitney–Wilcoxon test. (C) RT-qPCR analysis of gene expression in the livers of mice injected with vehicle (PBS), dex, or S961. Livers were isolated from unfed or fed mice at ZT14. Data are presented as mean ± SEM (n = 8–12). Statistical significance compared to fed-veh is calculated using Student t test. ***p < 0.001. (D–G) Examples of H3K27Ac and GR occupancy near genes regulated by dex or S961 injection. Numerical values for panels A–C are available in S12 Data. Bed graphs in panels C–G are available at GEO: GSE119713. dex, dexamethasone; DHS, DNase hypersensitive site; GR, glucocorticoid receptor; H3K27Ac, histone 3 lysine 27 acetylation; RT-qPCR, reverse transcription quantitative PCR; ZT, Zietgeber time. (TIF) [file pbio.2006249.s006.tif]

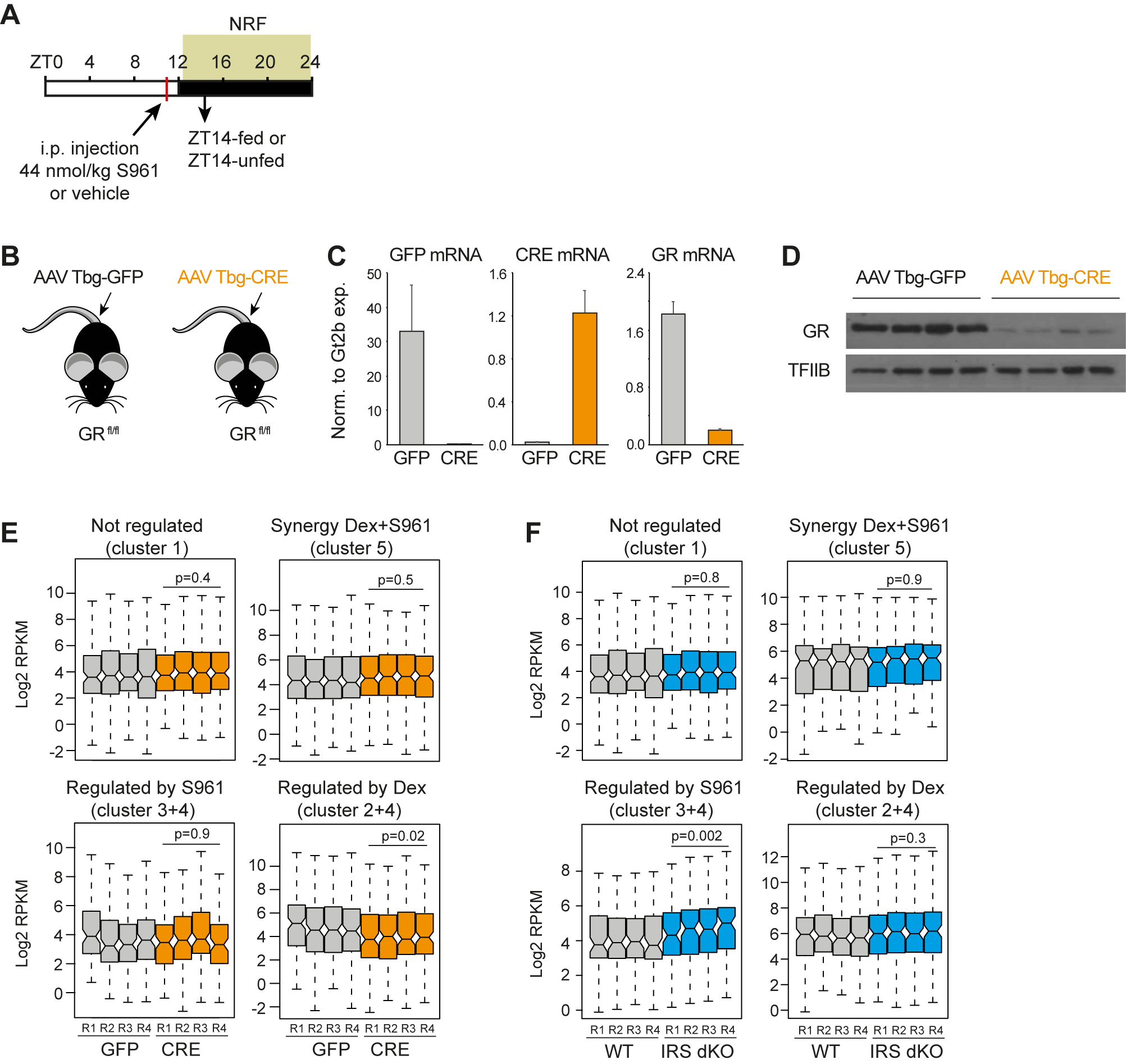

Supplement: S7 Fig — (A) Experimental setup of S961 injection and subsequent isolation of livers at ZT14. (B) Experimental setup of AAV–GFP and AAV–CRE injection of GRfl/fl mice. (C) RT-qPCR analysis of GFP, CRE, and GR expression two weeks post AAV injection. Data are presented as mean ± SEM (n = 4). (D) Western blot analysis of GR expression. (E) Quantification of mRNA expression (RNA-seq) in livers from GRfl/fl mice injected with AAV–GFP and AAV–CRE. Feeding-repressed genes regulated by dex, S961, or dex + S961 were analyzed separately. Log2 RPKM is visualized from four biological replicates (R1–R4). Statistical significance is calculated using a Mann–Whitney–Wilcoxon test of the mean of four biological replicates. (F) Quantification of mRNA expression (RNA-seq) in livers from WT and L-IRSdKO. Feeding-repressed genes regulated by dex, S961, or dex + S961 were analyzed separately. Log2 RPKM is visualized from four biological replicates (R1–R4). Statistical significance is calculated using a Mann–Whitney–Wilcoxon test of the mean of four biological replicates. Numerical values for panels B, C, E, and F are available in S13 Data. AAV, adeno-associated virus; CRE, CREB response element; dex, dexamethasone; GFP, Green fluorescent protein; GR, glucocorticoid receptor; L-IRSdKO, liver specific IRS double knockout; RPKM, reads per kilo base per million mapped reads; RT-qPCR, reverse transcription quantitative PCR; WT, wild-type; ZT, Zietgeber time. (TIF) [file pbio.2006249.s007.tif]

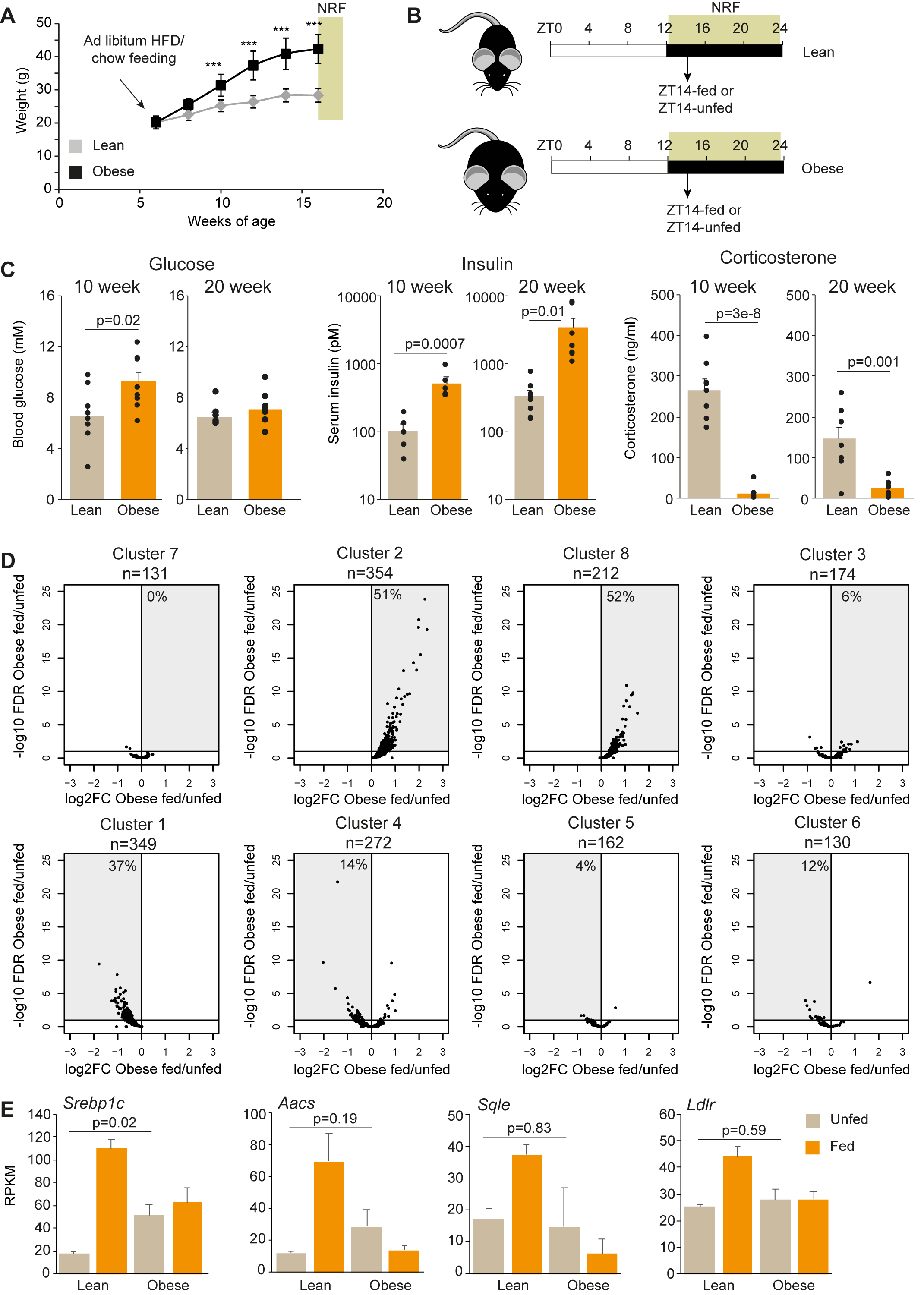

Supplement: S8 Fig — (A) Weight gain of diet induced obese animals compared to chow-fed controls. NRF was initiated 10 weeks after the start of HFD feeding. Chow fed (n = 16) and HFD fed (n = 16). (B) Experimental setup of NRF and indicated endpoints for the experiment. Livers were isolated at ZT14 from fed and unfed animals. (C) Preprandial (ZT14-unfed) blood was extracted from animals trained to night-restricted feeding at 10 and 20 weeks after initiation of HFD feeding. Glucose, insulin, and corticosterone were measured from at least five independent animals. Individual data points are marked in each panel. (D) Gene expression analysis of genes differentially expressed in obese animals. For each cluster, the percentage of differentially induced (cluster 1, 3, 7, and 8) and repressed genes (cluster 1, 4, 5, and 6) at FDR < 0.05 is indicated at the top. (E) Expression of Srebp1c and Srebp1c target genes (Aacs, Sqle, and Ldlr), measured by RNA-seq (n = 3). Data in panels A, C, and E are presented as mean ± SEM. Statistical significance is calculated using Student t test. *** indicates p < 0.001 or otherwise indicated in figure. Numerical values for panels A and C–E are available in S14 Data. FDR, false discovery rate; HFD, high-fat diet; NRF, night-restricted feeding; ZT, Zeitgeber time. (TIF) [file pbio.2006249.s008.tif]

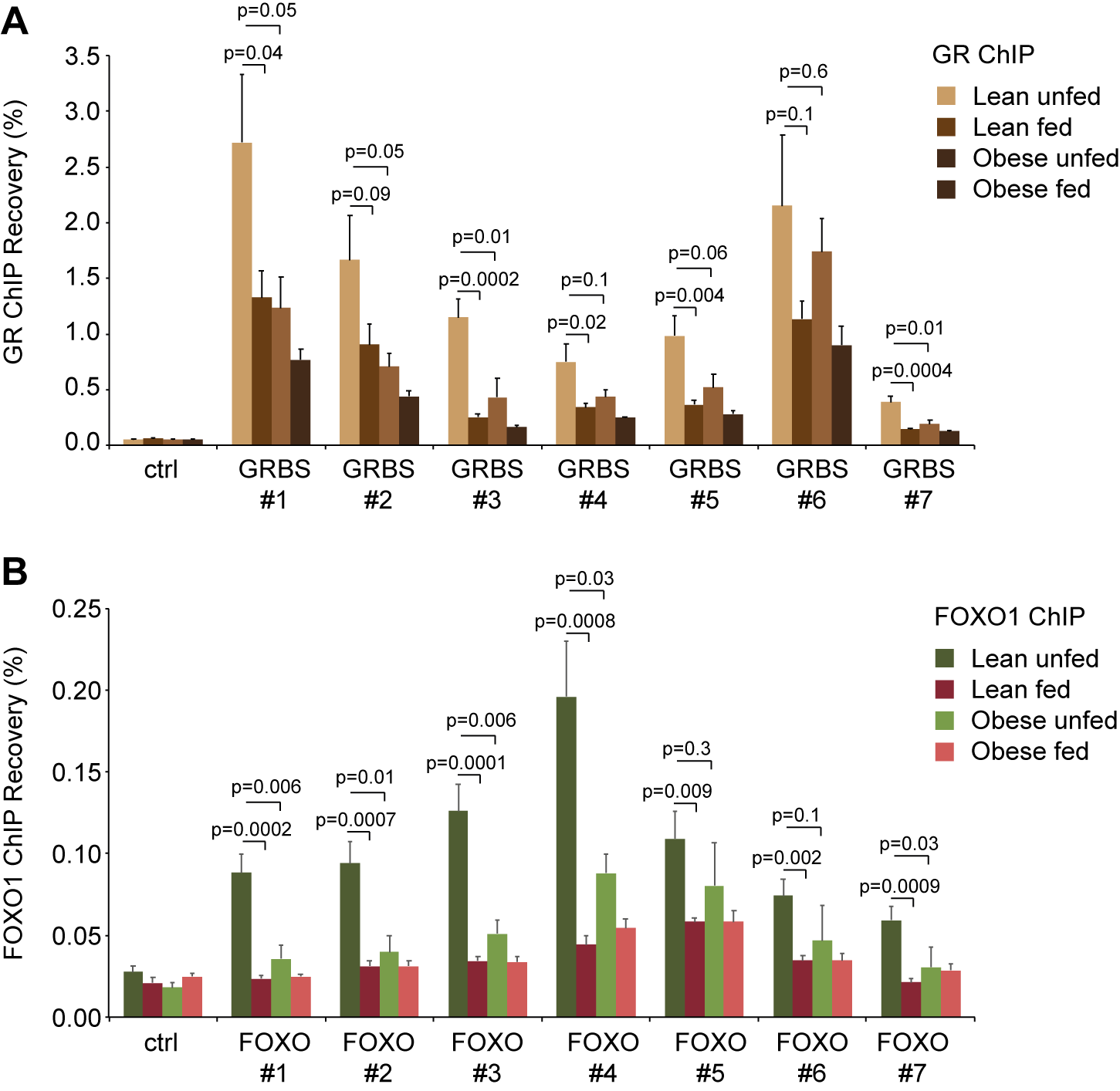

Supplement: S9 Fig — (A) Occupancy of GR at seven different GRBS (1–7) and one negative ctrl. (B) Occupancy of FOXO1 at seven different FOXO-binding sites (FOXO #1–7) and one negative (ctrl). Data are presented as mean ± SEM (n = 5–6). Statistical significance is calculated using Student t test. Numerical values for panels are available in S15 Data. ctrl, control site; FOXO1, Forkhead box O1; GR, glucocorticoid receptor; GRBS, GR binding site. (TIF) [file pbio.2006249.s009.tif]
