# Supplementary material for: Insulin signaling and reduced glucocorticoid receptor activity attenuate postprandial gene expression in liver
Source: PLoS Biol. 2018 Dec 10;16(12):e2006249. doi: 10.1371/journal.pbio.2006249 (PMC6301715; doi:10.1371/journal.pbio.2006249)
Supplement: S1 Table — (PDF) [file pbio.2006249.s010.pdf]

S1 Table. Illumina sequencing summary

Treatment, Genotype, Replicate	Type of experiment	Uniquely aligned reads	Data used in fig.
ZT10 unfed, single mouse, rep1	RNA-seq	27,275,587	Fig. 1
ZT10 unfed, single mouse, rep2	RNA-seq	30,894,006	Fig. 1
ZT14 unfed, single mouse, rep1	RNA-seq	38,423,813	Fig. 1
ZT14 unfed, single mouse, rep2	RNA-seq	33,027,220	Fig. 1
ZT14 fed, single mouse, rep1	RNA-seq	29,285,940	Fig. 1
ZT14 fed, single mouse, rep2	RNA-seq	27,275,587	Fig. 1
ZT10 unfed, single mouse, rep1	H3K27Ac ChIP-seq	18,813,773	Fig. 3
ZT10 unfed, single mouse, rep2	H3K27Ac ChIP-seq	13,365,436	Fig. 3
ZT10 unfed, single mouse, rep3	H3K27Ac ChIP-seq	16,881,214	Fig. 3
ZT14 unfed, single mouse, rep1	H3K27Ac ChIP-seq	17,124,365	Fig. 2 and 3
ZT14 unfed, single mouse, rep2	H3K27Ac ChIP-seq	16,682,113	Fig. 2 and 3
ZT14 unfed, single mouse, rep3	H3K27Ac ChIP-seq	17,065,873	Fig. 2 and 3
ZT14 fed, single mouse, rep1	H3K27Ac ChIP-seq	15,975,909	Fig. 2 and 3
ZT14 fed, single mouse, rep2	H3K27Ac ChIP-seq	17,799,136	Fig. 2 and 3
ZT14 fed, single mouse, rep3	H3K27Ac ChIP-seq	14,761,023	Fig. 2 and 3
ZT14 unfed, liver pooled from 3-4 mice, rep1	DHS-seq, 60U DNase	19,252,587	Fig. 2 and 3
ZT14 unfed, liver pooled from 3-4 mice, rep1	DHS-seq, 80U DNase	15,183,112	Fig. 2
ZT14 fed, liver pooled from 3-4 mice, rep1	DHS-seq, 60U DNase	16,567,900	Fig. 2
ZT14 fed, liver pooled from 3-4 mice, rep1	DHS-seq, 80U DNase	16,088,708	Fig .2
ZT14 unfed, liver pooled from 3-4 mice, rep2	DHS-seq, 60U DNase	10,279,844	Fig. 2
ZT14 unfed, liver pooled from 3-4 mice, rep2	DHS-seq, 80U DNase	11,333,928	Fig. 2
ZT14 fed, liver pooled from 3-4 mice, rep2	DHS-seq, 60U DNase	12,389,280	Fig. 2
ZT14 fed, liver pooled from 3-4 mice, rep2	DHS-seq, 80U DNase	12,322,369	Fig. 2
ZT14 unfed, liver pooled from 3-4 mice, rep1	GR ChIP-seq	22,551,189	Fig. 4
ZT14 unfed, liver pooled from 3-4 mice, rep2	GR ChIP-seq	12,646,858	Fig. 4
ZT14 fed, liver pooled from 3-4 mice, rep1	GR ChIP-seq	23,171,661	Fig. 4
ZT14 fed, liver pooled from 3-4 mice, rep2	GR ChIP-seq	11,755,324	Fig. 4
ZT14 unfed, liver pooled from 3-4 mice, rep1	ChIP input	22,614,848	Fig. 4
ZT14 fed, liver pooled from 3-4 mice, rep2	ChIP input	19,975,109	Fig. 4
ZT14 unfed, liver pooled from 3 mice, rep1	FoxO1 ChIP-seq	12,183,810	Fig. 4
ZT14 unfed, liver pooled from 3 mice, rep2	FoxO1 ChIP-seq	12,655,276	Fig. 4
ZT14 fed, liver pooled from 3 mice, rep1	FoxO1 ChIP-seq	17,289,263	Fig. 4
ZT14 fed, liver pooled from 3 mice, rep2	FoxO1 ChIP-seq	12,547,455	Fig. 4
ZT14 unfed PBS, liver pooled from 3-4 mice, rep1	GR ChIP-seq	8,307,810	Fig. 5
ZT14 Fed PBS, liver pooled from 3-4 mice, rep1	GR ChIP-seq	10,120,089	Fig. 5
ZT14 Fed Dex (1mg/kg), liver pooled from 3-4 mice, rep1	GR ChIP-seq	9,911,049	Fig. 5
ZT14 unfed PBS, liver pooled from 3-4 mice, rep1	H3K27Ac ChIP-seq	9,464,480	Fig. 5
ZT14 fed PBS, liver pooled from 3-4 mice, rep1	H3K27Ac ChIP-seq	8,679,768	Fig. 5
ZT14 Fed Dex (1mg/kg), liver pooled from 3-4 mice, rep1	H3K27Ac ChIP-seq	13,831,280	Fig. 5
ZT14 unfed PBS, liver pooled from 3-4 mice, rep2	H3K27Ac ChIP-seq	19,715,622	Fig. 5
ZT14 fed PBS, liver pooled from 3-4 mice, rep2	H3K27Ac ChIP-seq	16,859,732	Fig. 5
ZT14 Fed Dex (1mg/kg), liver pooled from 3-4 mice, rep2	H3K27Ac ChIP-seq	17,407,534	Fig. 5
ZT14 fed PBS, single mouse, rep1	RNA-seq	11,827,207	Fig.6
ZT14 fed PBS, single mouse, rep2	RNA-seq	10,275,936	Fig.6
ZT14 fed PBS, single mouse, rep3	RNA-seq	12,533,989	Fig.6
ZT14 fed PBS, single mouse, rep4	RNA-seq	9,218,670	Fig.6
ZT14 fed Dex (1mg/kg), single mouse, rep1	RNA-seq	10,856,686	Fig.6
ZT14 fed Dex (1mg/kg), single mouse, rep2	RNA-seq	6,501,158	Fig.6
ZT14 fed Dex (1mg/kg), single mouse, rep3	RNA-seq	10,825,727	Fig.6
ZT14 fed Dex (1mg/kg), single mouse, rep4	RNA-seq	8,686,099	Fig.6
ZT14 fed S961 (44 nM/mouse), single mouse, rep1	RNA-seq	12,023,461	Fig.6
ZT14 fed S961 (44 nM/mouse), single mouse, rep2	RNA-seq	9,402,575	Fig.6
ZT14 fed S961 (44 nM/mouse), single mouse, rep3	RNA-seq	13,541,304	Fig.6
ZT14 fed S961 (44 nM/mouse), single mouse, rep4	RNA-seq	10,186,675	Fig.6
ZT14 fed S961 (44 nM/mouse) + Dex (1mg/kg), single mouse, rep1	RNA-seq	11,498,424	Fig.6
ZT14 fed S961 (44 nM/mouse) + Dex (1mg/kg), single mouse, rep2	RNA-seq	9,911,870	Fig.6
ZT14 fed S961 (44 nM/mouse) + Dex (1mg/kg), single mouse, rep3	RNA-seq	10,707,806	Fig.6
ZT14 fed S961 (44 nM/mouse) + Dex (1mg/kg), single mouse, rep4	RNA-seq	8,077,668	Fig.6
ZT14 unfed PBS, single mouse, rep1	RNA-seq	12,466,250	Fig.6
ZT14 unfd PBS, single mouse, rep2	RNA-seq	10,886,948	Fig.6
ZT14 unfed PBS, single mouse, rep3	RNA-seq	10,574,048	Fig.6
ZT14 unfed PBS, single mouse, rep4	RNA-seq	10,285,997	Fig.6
ZT14 unfed, single mouse (GR fl/fl), AAV-GFP, rep1	RNA-seq	17,505,049	Fig.6
ZT14 unfed, single mouse (GR fl/fl), AAV-GFP, rep2	RNA-seq	15,790,803	Fig.6

ZT14 unfed, single mouse (GR fl/fl), AAV-GFP, rep3	RNA-seq	19,830,161	Fig.6
ZT14 unfed, single mouse (GR fl/fl), AAV-GFP, rep4	RNA-seq	28,122,661	Fig.6
ZT14 unfed, single mouse (GR fl/fl), AAV-CRE, rep1	RNA-seq	26,733,544	Fig.6
ZT14 unfed, single mouse (GR fl/fl), AAV-CRE, rep2	RNA-seq	19,656,406	Fig.6
ZT14 unfed, single mouse (GR fl/fl), AAV-CRE, rep3	RNA-seq	22,185,956	Fig.6
ZT14 unfed, single mouse (GR fl/fl), AAV-CRE, rep4	RNA-seq	17,373,726	Fig.6
ZT14 fed Dex (1mg/kg), single mouse, WT, rep1	RNA-seq	7,548,005	Fig.6
ZT14 fed Dex (1mg/kg), single mouse, WT, rep2	RNA-seq	8,216,998	Fig.6
ZT14 fed Dex (1mg/kg), single mouse, WT, rep3	RNA-seq	7,774,419	Fig.6
ZT14 fed Dex (1mg/kg), single mouse, WT, rep4	RNA-seq	8,703,002	Fig.6
ZT14 fed Dex (1mg/kg), single mouse, L-IRS1/2 dKO, rep1	RNA-seq	7,988,596	Fig.6
ZT14 fed Dex (1mg/kg), single mouse, L-IRS1/2 dKO, rep2	RNA-seq	8,974,250	Fig.6
ZT14 fed Dex (1mg/kg), single mouse, L-IRS1/2 dKO, rep3	RNA-seq	8,036,663	Fig.6
ZT14 fed Dex (1mg/kg), single mouse, L-IRS1/2 dKO, rep4	RNA-seq	9,551,713	Fig.6
ZT14 unfed, HFD single mouse, rep1	RNA-seq	27,440,265	Fig.7
ZT14 unfed, HFD single mouse, rep2	RNA-seq	21,413,176	Fig.7
ZT14 unfed, HFD single mouse, rep3	RNA-seq	24,266,317	Fig.7
ZT14 unfed, chow diet, single mouse, rep1	RNA-seq	26,607,515	Fig.7
ZT14 unfed, chow diet, single mouse, rep2	RNA-seq	18,612,208	Fig.7
ZT14 unfed, chow diet, single mouse, rep3	RNA-seq	16,968,978	Fig.7
ZT14 fed, HFD, single mouse, rep1	RNA-seq	62,579,548	Fig.7
ZT14 fed, HFD, single mouse, rep2	RNA-seq	17,357,688	Fig.7
ZT14 fed, HFD, single mouse, rep3	RNA-seq	20,119,313	Fig.7
ZT14 fed, chow diet, single mouse, rep1	RNA-seq	19,005,758	Fig.7
ZT14 fed, chow diet, single mouse, rep2	RNA-seq	21,172,006	Fig.7
ZT14 fed, chow diet, single mouse, rep3	RNA-seq	22,669,394	Fig.7
