# Supplementary material for: Insulin signaling and reduced glucocorticoid receptor activity attenuate postprandial gene expression in liver
Source: PLoS Biol. 2018 Dec 10;16(12):e2006249. doi: 10.1371/journal.pbio.2006249 (PMC6301715; doi:10.1371/journal.pbio.2006249)
Supplement: S2 Table — qPCR, quantitative PCR. (PDF) [file pbio.2006249.s011.pdf]

S2 Table. Primers used for qPCR analysis

Gene name	5' primer	3'primer
Bmal1	GCCAGCCCAGCACTAACACTCAC	TCTTCGTCCAGCCCCATCTTCGT
Reverba	TCCTGACACGGTTCTCGCCCT	GGCGCTCTGCATCTCGGCAA
Cry1	GGATCCACCATTTAGCCAGA	CTCCTGCAAATGCTACGTCA
Dbp	CGGCTCCCGTCTCCTCACTT	GCAGAGTTGCCTTGCGCTCCT
Gtf2b (TFIIB)	GTTCTGCTCCAACCTTTGCCT	TGTGTAGCTGCCATCTGCACTT
Insig2	TGGCTTCCTTGTTTCTGATGGCTCC	ACAGTGCAGCCAGTGTGAGGGA
Angptl4	GCCCCGCCTCCAATGCTCTC	GTTTCACGGTTGCACCTAAAGCCCC
G6Pc	TCGGAGACTGGTTCAACCTC	CTCAAGCCAATGGGGACTTA
TAT	TGAGATTCGGTTGCCCTGGAGA	CCGGCTACAAGCCTCCAGCATC
Fkbp5	AGTGCTGAGGGGTGGCTTGT	TGTCCTTCCACCACAGCAGCC
Pck1	ACATTGCCTGGATGAAGTTTGAT	GGTTGATAGCCCTTAAGTTGCCTT
**GR binding site (GRBS)**		
GRBS #1	AGCCCTTTGGCCTCTAGAAC	AATGTCACCAGGGCAGTCTC
GRBS #2	ATAAAATGCGCAGGATGAGG	TTGTCTCCAGGCTGAACTCC
GRBS #3	GGATCGAGGATGACCTGTGT	CCAGGAGACAAATGTCTTGGA
GRBS #4	GGGACAAATGAGGCAAAAGA	GGCTTTCTTCATCGACAAGC
GRBS #5	CGAAGCGGCATATTTGAGAT	TCAGGATCATCTGCCAACAA
GRBS #6	TCAACTGCAGCAAAGAGAGC	GAGGCAGGTTCATTTGAGGA
GRBS #7	TCACAGGGATTGTGTGCTGT	ACAGAGAGCCCCATGTTCAC
**FOXO binding site**		
FOXO #1	CTCTATCGCAGGGAGAGCTG	GTTTGCGGGATGGTTTTCTA
FOXO #2	TTGCGAGCTTACATCACCAG	AGCCAGCTAAATGGCAGAGA
FOXO #3	TTTCTCTGGCTGCTCTCACA	CTGTCCTTAGGTGGCTCTGG
FOXO #4	GTCACGCACTCTAGGCAACA	GGCGTGGAGAGAAAAATCAG
FOXO #5	TGTTTAGGGCAAAGGTTGGT	TTGGCCTTGTTTACGTAGGG
FOXO #6	TGGATCATCGCTCAACAAAG	CCACAGTGACCTGGTTTCCT
FOXO #7	CACCTGGCTGCTAAACATCA	GAGGCATTTGGATTTGTCGT
**Control site**		
No GR and FOXO binding	TGGTAGCCTCAGGAGCTTGC	ATCCAAGATGGGACCAAGCTG
